# Integrated systemic analysis of FAM72A to identify its clinical relevance, biological function, and relationship to drug sensitivity in hepatocellular carcinoma

**DOI:** 10.3389/fonc.2022.1046473

**Published:** 2022-11-22

**Authors:** Qi Zhou, Lingjun Chen, Luo Yang, Haoxiong Zhou, Yan Chen, Yunwei Guo

**Affiliations:** ^1^ Department of Gastroenterology, Third Affiliated Hospital, Sun Yat-sen University, Guangzhou, China; ^2^ Department of Infectious Diseases, Third Affiliated Hospital, Sun Yat-sen University, Guangzhou, China

**Keywords:** FAM72, HCC, prognosis, FAM72A, mTOR, immunotherapy, biomarker

## Abstract

**Background:**

The family with sequence similarity 72 member A (FAM72A) protein has been identified as an effector of multiple pathological processes in many cancers. The value of FAM72A in HCC remains largely unknown.

**Methods:**

Data from TCGA-LIHC, ICGC-LIRI-JP, IMvigor210, cBioPortal, GeneMANIA, and TIMER were processed and visualized to explore the association between FAM72A and the prognosis, stemness phenotype, mutational burden, immune cell infiltration, and drug sensitivity in HCC patients. Potential pathways were also revealed. Furthermore, we experimentally verified the results *in vivo* and *in vitro* using immunohistochemistry, western blotting, and CCK-8 assays.

**Results:**

First, FAM72A mRNA expression was significantly upregulated in HCC. High FAM72A expression was independently associated with a poor prognosis. Experimental validation confirmed that FAM72A was remarkably overexpressed in HCC patients and mice. Moreover, FAM72A knockdown suppressed HCC cell proliferation. In addition, the frequency of TP53 mutations was significantly higher in the high FAM72A expression group. Subsequently, the enrichment analysis revealed that FAM72A was closely related to immune processes and mTOR pathways. Silencing FAM72A increased the expression levels of mTOR in HCC cell lines. The FAM72A-mTOR pathway was strongly associated with a poor prognosis for patients with HCC. Patients with high FAM72A expression levels might be more resistant to sorafenib. Furthermore, the expression of FAM72A and mTOR was significantly associated with the abundance of some tumor-infiltrating immune cells, especially CD4^+^ T cells. Finally, patients with high levels of FAM72A and mTOR were more sensitive to immunotherapy.

**Conclusions:**

FAM72A, a member of the FAM72 family, might be a prognostic and immunotherapeutic target for HCC patients.

## Introduction

Hepatocellular carcinoma (HCC), arising from hepatocytes, is a leading cause of cancer-related deaths worldwide (1). HCC has a poor prognosis, as evidenced by 5-year survival rates ranging from 4% to 17% (2). Early-stage HCC is asymptomatic, causing considerable challenges to intermediate diagnosis and effective treatment. Based on accumulating evidence, immune checkpoint inhibitors (ICIs) are indicated as a standard of cancer treatment, not only for metastasized malignancies (3) but also for early-stage tumors (4). However, the immune microenvironment of HCC is highly heterogeneous (5). A clinical assessment of tumor heterogeneity is required to facilitate the development of more effective personalized therapy. Therefore, the identification of effective targets for the early diagnosis, individualized immunotherapy, and prognostic assessments of HCC is essential to improve the survival rate of patients.

Family with sequence similarity 72 member A-D (FAM72A-D) is a novel protein-coding gene expressed in neural stem cells (6). The FAM72 protein has been identified to be involved in neuronal signaling pathways (7) and maintains the self-renewal capacity of neuronal progenitor cells (8) in the brain. However, under pathophysiological conditions, FAM72 may lead to postmitotic neuronal cell death (6) and play a pivotal role in various cancers (6, 9–11). However, the diagnostic and prognostic values of different FAM72 members in HCC remain unclear, and the association of the FAM72 family with the HCC microenvironment has not been reported to date. Notably, humans express four FAM72 family genes. In contrast, all other species express only one gene specifically (5, 12). A previous study reported that high FAM72A expression reduces UNG2 levels, leading to an increased susceptibility of cells to mutations, and error-free DNA repair continues into S phase (13). Under normal physiological conditions, FAM72A promotes mutagenic repair during antibody maturation through activation-induced cytidine deaminase (AID) (13, 14). Uncontrolled mutations in antibody-producing B cells are associated with B cell lymphoma (14). In addition, FAM72A is expressed at high levels in other cancers, such as gastrointestinal, breast, lung, liver, and ovarian cancers (9, 15–19). High levels of FAM72A may promote mutations in antibody-related genes, and therefore, increased levels of FAM72A may stimulate cancer development, progression, or drug resistance by increasing the number of mutations. The biological function of FAM72A in HCC remains to be further investigated.

In the present study, we harnessed a multiomics database to comprehensively verify the prognostic and biological significance of FAM72A in HCC. First, considering the limited knowledge of the function of FAM72 proteins in cancers, we investigated its relationship with genetic changes, prognosis, stemness and the tumor microenvironment (TME) across cancer types. Then, we evaluated the differences in expression and diagnostic and prognostic values of FAM72 members for HCC patients. We subsequently assessed the association between FAM72A expression and stemness and gene alterations. Furthermore, we predicted a potential downstream pathway linked to shorter overall survival (OS) of HCC patients. Finally, we evaluated the levels of immune cell infiltration and immune checkpoint expression. We propose that this study may contribute to a better comprehension of the molecular mechanisms involved in the pathophysiological processes of HCC and ultimately facilitate the development of new therapeutic strategies.

## Materials and methods

### Data acquisition and processing

In our analysis, the Fragments Per Kilobase per Million (FPKM) read-based gene expression data and clinical information of patients with 33 types of cancer were obtained from UCSC Xena (http://xena.ucsc.edu/). In addition, we downloaded two liver hepatocellular carcinoma (LIHC) transcriptomic datasets and matched clinical data. TCGA-LIHC dataset with 374 HCC and 50 normal samples was retrieved from the Genomic Data Commons (GDC) Data Portal (https://portal.gdc.cancer.gov/). Two hundred forty HCC samples and 197 normal samples from the ICGC portal (ICGC-LIRI-JP, https://dcc.icgc.org/projects/LIRI-JP) were also included. The procedure is shown in **Supplementary Figure 1**.

### mRNA expression levels and interaction network analysis

We compared the expression of FAM72 family genes between 18 types of tumor and normal tissues (normal samples ≥ 5) using the Wilcoxon test. The “limma” R package was also used to compare the difference in FAM72A-D expression between HCC and normal samples after setting thresholds as p < 0.05 and |log2FC| (FC: fold change) > 1. Moreover, we predicted the correlations among FAM72 family genes using the “corrplot” R package. The GeneMANIA database was also introduced to develop a potential network of interaction functions. Furthermore, we selected the top 100 highly correlated genes with high gene expression to create the active gene set and scored the samples according to their expression. The Wilcoxon test assessed differences in scores between normal and tumor tissues.

### Analysis of prognostic and diagnostic value

Cox regression analysis was conducted to select genes independently associated with overall survival (OS) among the FAM72 family genes. We visualized the results using the “survival” and “forestplot” R packages. The receiver operating characteristic (ROC) curve was analyzed to determine the accuracy of FAM72 family members in diagnosing tumors. The area under the curve (AUC) was calculated to reflect the efficacy of the diagnosis. According to the median FAM72 expression, TCGA-LIHC cohort was divided into FAM72-high and FAM72-low groups. The Kaplan−Meier log-rank test was subsequently applied to evaluate whether high FAM72A expression levels were associated with shorter OS for HCC patients stratified into different clinical subgroups.

### Stemness analysis

We used the mRNA-si stemness score (20) and other stemness signatures [Ben-Porath ESC score (21), Wong ESC score (22), and Bhattacharya ESC score (23)] to assess the relationship between FAM72A and stemness. For each stage and grade, PCNA expression was extracted from TCGA-LIHC database to investigate the relationship with FAM72A expression. In addition, the proliferation score was obtained from previous studies. Each patient was scored to assess the capacity of FAM72A to predict the proliferative potential.

### Genomic alterations and mutation profiles

The mutation frequency of different FAM72 family genes in various TCGA cancer types was analyzed using the cBioPortal database (http://www.cbioportal.org/). Variations in FAM72 family members were calculated using the Consortium Human build 38 (GRCh38) genome date. The mutation comment file (MAF) of TCGA-LIHC cohort was also downloaded from the GDC client and visualized with the “martfool” R package to further explore the effect of different FAM72A expression levels on gene mutation. Moreover, the relationships between FAM72A levels and the number of segments, aneuploid core, and fraction altered score (24) were evaluated by performing “Spearman’s” correlation analysis.

### Prediction of potential axes involved in HCC

Gene set enrichment analysis (GSEA) was also performed for potential downstream pathways using GSEA software (version 4.2.1) (http://www.gsea-msigdb.org). Next, “c2.cp.kegg.v7.4.symbols.gmt” was used to detect pathways and molecular mechanisms. Considering the gene expression profile of the two groups, the minimum gene set was set to 15, and the maximum was set to 500. p < 0.05 and FDR < 0.25 were considered statistically significant. The correlation between the expression of key genes in potential candidate pathways and FAM72A expression was subsequently evaluated using Spearman’s correlation analysis based on TCGA-LIHC data. The differential expression of central genes and their effects on survival were subsequently studied.

### Gene Ontology (GO) and Kyoto Encyclopedia of Genes and Genomes (KEGG) enrichment analyses

First, the Wilcoxon test was utilized to identify the differentially expressed genes (DEGs) between the high and low FAM72A expression groups with thresholds of p < 0.05 and |log2FC| > 0.1. DEGs that were upregulated in the high FAM72A expression group were selected for subsequent KEGG and GO enrichment analyses using the “ClusterProfiler” R package to explore the underlying functional annotations.

### Analysis of immune-related parameters

The “ESTIMATE” R package was used to calculate the immune and stromal scores for various types of cancer (25). The “GSVA” R package was applied to quantify the abundance of 29 immune cell types in TCGA-LIHC cohort. We also used the CIBERSOFT algorithm to evaluate the proportions of 22 immune cell types in samples stratified by FAM72A expression. In addition, the correlations between FAM72A expression levels and immune scores [intratumor heterogeneity, leukocyte fraction, and TGF-β response (24)] and immune cells (macrophages M0, Th2 cells, and Th17 cells) were assessed by performing Spearman’s correlation analysis. Furthermore, the “Gene_Corr” module of the TIMER2.0 database (http://timer.cistrome.org/) was utilized to explore the correlation between mTOR expression and immune cell infiltrates. Afterward, we extracted data for specific immune checkpoint genes to further explore whether gene expression levels correlated with immunotherapy. The IMvigor210 cohort (http://research-pub.Gene.com/imvigor210corebiologies) was then applied to predict the potential of patients with different gene expression levels to respond to anti-PD-L1 antibody (pembrolizumab).

### Drug sensitivity analysis

The “oncoPredict” R package was used to predict the relationship between FAM72A gene expression levels and drug sensitivity. Notably, oncoPredict fits approximately 20,000 gene expression profiles from 809 cell lines to the half-maximal inhibitory concentration (IC50) values for 198 drugs. Drugs were obtained from Genomics of Cancer Drug Sensitivity (GDSC; https://www.cancerrxgene.org/), and gene expression profiles for cancer cell lines were obtained from the Boulder Institute Cancer Cell Line Encyclopedia (CCLE; https://portals.broadinstitute.org/ccle_legacy/home). The lower the IC50 value, the more sensitive patients are to the drugs, helping guide patients’ clinical medications.

### Mouse model of HCC

Animal research protocols were approved by the Institutional Animal Care and Use Committee at the Third Affiliated Hospital of Sun Yat-sen University. C57BL/6 male mice (15 days old, Guangdong GemPharmatech Co., Ltd.) were randomized into two groups. Mice in the experimental group were intraperitoneally injected with 15 mg/kg DEN (Sigma, N0756) and an equal amount of saline was intraperitoneally injected into the control group. Five mice were allocated to each of the groups. The mice were housed in specific pathogen−free (SPF) environments in microisolator cages on a 12-hour dark-light cycle (lights on at 8:00 a.m.) at a temperature of 20–25 °C with food and water provided ad libitum. Ketamine (50 mg/kg) was injected intraperitoneally as the anesthetic. Carbon dioxide inhalation was used as the method for euthanasia. These mice were sacrificed after nine months.

### Patient samples

Three paired HCC and paracancerous tissues (> 2 cm for the edge of tumors) were obtained during operations on *de novo* HCC patients. All experiments were performed according to the principles of the Declaration of Helsinki. The protocol was approved by the Research Ethics Committee of The Third Affiliated Hospital of Sun Yat‐sen University. Written informed consent was obtained from all patients.

### Immunohistochemical (IHC) staining

Hematoxylin and eosin (H&E) staining and immunohistochemistry were performed on both mouse and human tissues using a previously published protocol (26). Paraffin sections (three-micrometer) were deparaffinized, rehydrated, and subjected to antigen retrieval and blocking (3% H_2_O_2_, 5% goat serum). A FAM72A (Proteintech, anti-rabbit, 21203-1-AP) monoclonal antibody was used to detect the levels of this protein. A semiquantitative analysis of the histological staining was performed using ImageJ software.

### Cell culture and treatment

HCC cell lines, including HepG2 (HB-8065) and Hep3B (HB-8064) cells, were obtained from the American Type Culture Collection and maintained in Dulbecco’s modified Eagle’s medium (Gibco BRL, Rockville, MD, USA) supplemented with 10% fetal bovine serum (Gibco BRL) at 37°C with 5% CO_2_. HA-FAM72A was cloned into the pcDNA3.1 vector. siFAM72A1 (5′- GCAGUGGACUUCACUGGAATT−3′) and siFAM72A2 (5′- CCUGCAACAACGGACACUUTT −3′) were purchased from GenePhama (Shanghai, China). Transient plasmid or siRNA transfection was conducted using Lipofectamine 3000 transfection reagent (Invitrogen, Carlsbad, CA, USA). HepG2 and Hep3B cell lines were harvested 48** **h after transfection for further analysis. For drug intervention, both HCC cell lines were treated with 20 μM rapamycin (MCE, HY-10219) or 5 μM sorafenib (MCE, HY-10201S2).

### Western blotting analysis

Western blotting was performed on paraffin sections as described in our previous study (27). Briefly, tissues and cells were lysed using RIPA buffer, and protein samples were quantified using a BCA protein assay kit (Macgene, MPK002) and boiled in 5× loading buffer. Then, total protein extracts from tissues and cells were analyzed using western blotting with antibodies against FAM72A (Proteintech, anti-rabbit, 21203-1-AP), mTOR (Cell Signaling Technology, anti-rabbit, mAb 2983), p-mTOR (1:1000, 5536, CST), PCNA (1:2000, 13110, CST), CCNE1/cyclin E1 (Abcam, ab71535), CCND1/cyclin D1 (Cell Signaling Technology, 55506), and CDK2 (Abcam, ab32147). An anti-β-actin antibody (Sigma, anti-rabbit, A5441) was used as the internal reference. Western blot results were quantified using ImageJ software.

### Cell Counting Kit-8 (CCK-8) assay

Cell counting assays were used to assess the proliferation of Hep3B and HepG2 cells ***in vitro***. Growing cells were plated in 96-well plates (1 × 10^3^/well). The cells were incubated in a cell incubator at 37°C with 5% CO_2_. Subsequently, 20 μl of CCK-8 (Dojindo, CK04) solution were added to 180 μl of culture medium. After 2** **h, the absorbance was measured at a wavelength of 450 nm. Experiments were performed in triplicate wells and three times independently.

### Statistical analysis

We used R software (version 4.2.1, http://www.R-project.org) and GraphPad Prism 8 software for statistical analyses. Measurement data are presented as the means ± standard errors. Univariate and multivariate Cox regression analyses were conducted using the “survival” package. The log-rank test was used to evaluate the differences in OS. Student’s t test, Wilcoxon test, one-way analysis of variance (ANOVA), or Chi-square test were utilized to evaluate the statistical significance. The Spearman correlation test was performed to generate the correlation matrix. p < 0.05 was considered statistically significant, and the p value was two-sided. Experiments were repeated three times at nonoverlapping time spans.

## Results

### The expression pattern of the FAM72 gene differs across cancers

Of the 33 cancer types obtained from the UCSC Xena platform, 18 tumors with a normal sample size greater than five were included in the subsequent differential expression analysis. The results of the pancancer analysis showed that FAM72 family genes were expressed at significantly higher levels in a variety of tumors than in the corresponding normal tissues (**Supplementary Figure 2A**). The expression profiles of FAM72 family genes were different in tumors (**Supplementary Figure 2B**). A significant positive correlation was observed among FAM72 family members (**Supplementary Figure 2C**). Then, a univariate analysis was performed to evaluate the prognostic value of FAM72 family genes. FAM72 family gene overexpression was significantly linked to poor outcomes, particularly in HCC (**Supplementary Figure 3**). FAM72 family genes may be helpful as prognostic indicators. By establishing a correlation test with RNAss scores, we observed a positive correlation between the high expression of the FAM72 family genes and stemness in most tumors, especially in HCC (**Supplementary Figure 4A**). We also obtained the frequency of FAM72A-D mutations using cBioPortal. The mutation frequencies of all four genes in HCC were higher than those in most tumors (**Supplementary Figure 4B**). Furthermore, we analyzed the correlations between FAM72 expression and tumor immunity to comprehensively understand whether FAM72 expression was related to tumor immunity. In the C2 immune subtype, the expression of FAN72 genes was significantly increased (**Supplementary Figure 5A**). As essential indicators for evaluating the tumor microenvironment (TME), the stromal and immune scores were significantly correlated with FAM72 gene expression in most cancers, including HCC (**Supplementary Figures 5B, C**).

### FAM72 is expressed at high levels in HCC and correlates with the cell cycle

**Figure 1:**
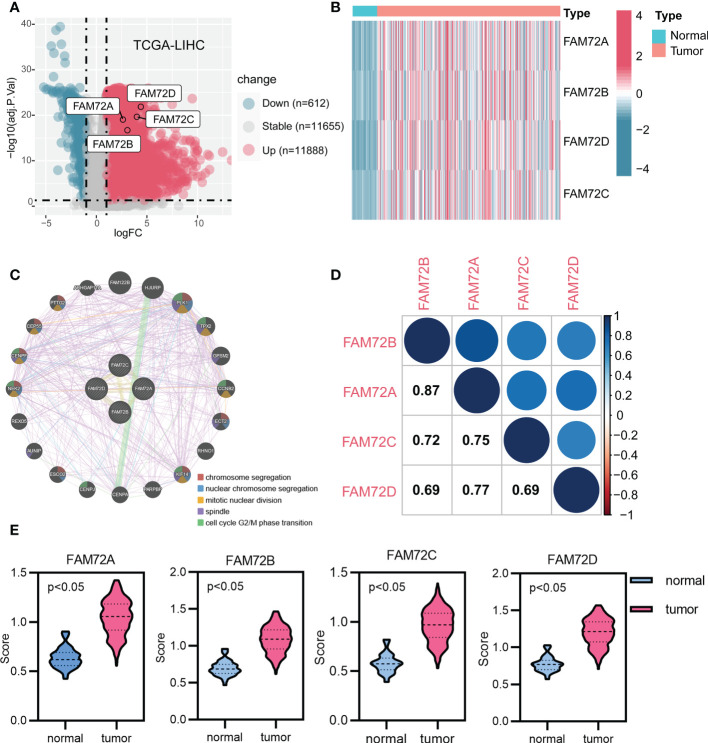
Gene expression and interaction network of FAM72 family members in TCGA-LIHC cohort. **(A)** Volcano plot of expression profiles. **(B)** Heatmap of expression profiles. **(C)** The gene network analyzed using the GeneMANIA database. **(D)** Correlation analysis among FAM72 family members. Statistical analyses were performed using the Spearman correlation test. **(E)** Comparison of activity scores between the normal and tumor groups. Statistical analyses were performed using the Wilcoxon test.

Considering the limited knowledge surrounding the function of the FAM72 family in HCC, we further investigated the association of FAM72 family genes with HCC using multiple databases. First, we analyzed the differential expression of FAM72 genes. All FAM72 family genes were expressed at significantly higher levels in HCC (**Figures 1A, B**). In addition, a gene network analysis based on the GeneMANIA database indicated that the FAM72 family was interrelated with 20 potential target genes. Their interaction functions mainly involved chromosomal and cell cycle alterations (**Figure 1C**). Afterward, we evaluated the correlations among FAM72 family genes and observed strong positive correlations with other family members that were involved in cell cycle alterations (**Figure 1D**). In addition, we further evaluated the distribution of FAM72 activity scores in both normal and tumor samples. FAM72 activity scores were significantly higher in HCC (**Figure 1E**).

### FAM72A is upregulated in HCC and correlates with shorter survival

**Figure 2:**
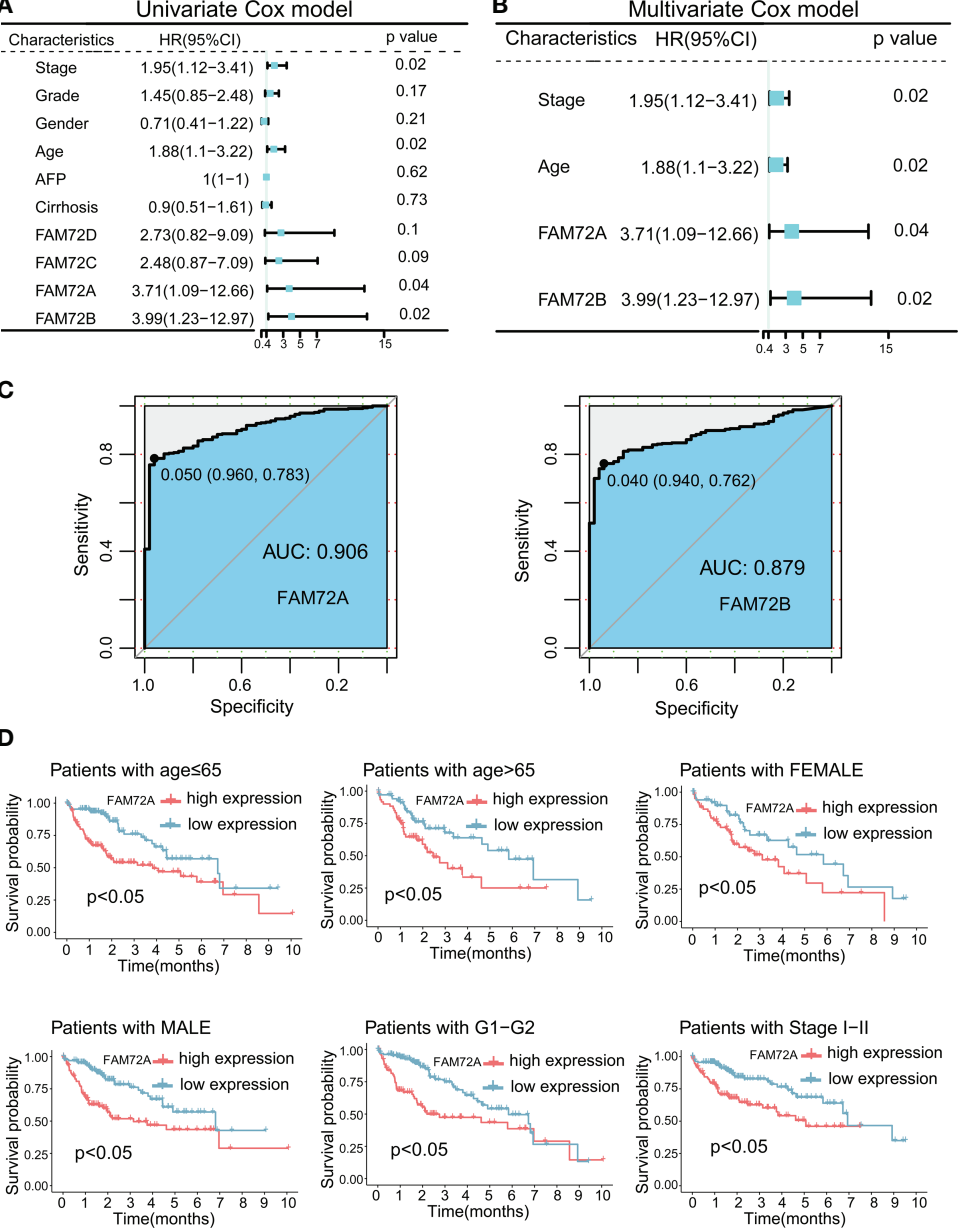
Prognostic and diagnostic significance of FAM72 family genes in TCGA-LIHC cohort. **(A)** Univariate Cox regression analyses for predicting the OS of HCC patients. **(B)** Multivariate Cox regression analyses for predicting the OS of HCC patients. Statistical analyses were performed using the Wilcoxon test. **(C)** ROC curve analysis of the diagnostic values of FAM72A and FAM72B. **(D)** Kaplan−Meier survival curves comparing the high and low expression of FAM72A in the patients stratified by different clinicopathological characteristics. Statistical analyses were performed using the log-rank test. HR, hazard ratio; OS, overall survival; HCC, hepatocellular carcinoma; ROC, receiver operating characteristic.

We first calculated the overall survival (OS) rate of HCC patients with high and low FAM72 expression to further explore the predictive role of FAM72 in the prognosis of HCC. The highest five-year survival rate was observed in the low FAM72A expression group (72.8%) among other FAM72 family genes (FAM72B: 72.7%; FAM72C: 69.8%; FAM72D: 67.9%) (**Supplementary Figure 6**). Then, we performed univariate and multifactorial Cox regression analyses. High expression levels of FAM72A and FAM72B were independent risk factors for OS (**Figures 2A, B**). Hence, FAM72A, B was picked for the following study. Subsequently, ROC curves were constructed to evaluate the diagnostic value of both FAM72A and FAM72B. FAM72A exhibited a better predictive power for diagnosing HCC, with an AUC of 0.906 (**Figure 2C**). FAM72A was included in subsequent studies as an independent prognostic gene in HCC patients with a high diagnostic value for HCC. Furthermore, we assessed the prognosis of patients in subgroups stratified according to different clinical characteristics. The Kaplan−Meier analysis confirmed that high FAM72A expression was strongly associated with a poor prognosis for elderly/nonelderly men and women. FAM72A might be a potential target for predicting the prognosis of patients with early-stage HCC (**Figure 2D**; **Supplementary Figure 7**). Similar results were obtained using the ICGC-LIRI-JP database (**Supplementary Figure 8**).

### FAM72A promotes HCC cell proliferation

**Figure 3:**
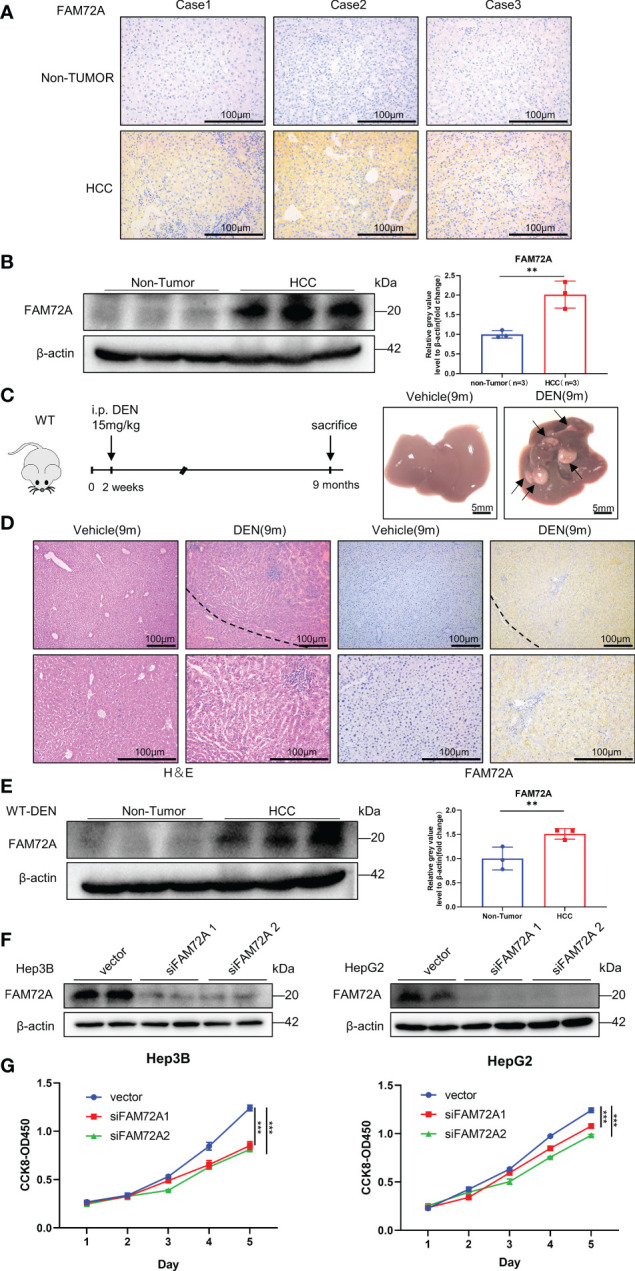
Experimental validation of FAM72A expression in HCC. **(A)** Representative images of FAM72A staining in human HCC and paracancerous tissues from each patient. Scale bar: 100 μm. **(B)** FAM72A protein expression in human HCC and paracancerous tissues from each patient was determined using western blotting. β-Actin was used as the loading control. Error bars represent the means ± SDs of triplicate experiments. Statistical analyses were performed using Student’s t test. **(C)** Photographs of the livers from WT (vehicle) and WT (DEN) mice at 9 months. Scale bar: 5 mm; 9 m: 9 months. **(D)** Representative images of H&E and FAM72A staining in HCC and normal control mice. Scale bar: 100 μm; 9 m: 9 months. **(E)** FAM72A protein expression in WT (vehicle) and WT (DEN) mouse liver tissues was determined using western blotting. β-Actin was used as the loading control. Error bars are presented as the means ± SDs of triplicate experiments. Statistical analyses were performed using Student’s t test. **(F)** Western blot analysis of FAM72A protein levels in Hep3B and HepG2 cell lines after FAM72A was knocked down. **(G)** CCK-8 analysis after FAM72A knockdown in the indicated cells. Error bars are presented as the means ± SDs of triplicate experiments. Statistical analyses were performed using one-way ANOVA. HCC, hepatocellular carcinoma; CCK-8, Cell Counting Kit-8. The aforementioned experiments were repeated three times. **p < 0.01, and ***p < 0.001.

We conducted some validation experiments to further substantiate the results from the bioinformatics analysis. FAM72A, as the most representative gene of the FAM72 family involved in HCC identified in the results described above, was selected for further experiments. According to our findings, FAM72A is functionally connected to cell cycle alterations. We first analyzed the relationship between FAM72A and cell cycle alterations in HCC cell lines. The levels of crucial regulatory proteins for the cell cycle, namely, CCNE1, CCND1, and CDK2, were inhibited by siFAM72A (**Supplementary Figures 9A, B**). Later, we further identified the expression levels of FAM72A in HCC patients and DEN-induced HCC mouse models by performing immunohistochemical staining and western blot analysis. FAM72A was expressed at high levels in HCC tissues from patients (**Figures 3A, B**; **Supplementary Figure 9C**). Similarly, in the successfully constructed HCC mouse model (**Figure 3C**), FAM72A was identified to be expressed at high levels in mouse tumor tissues at 9 months by performing immunohistochemical staining and western blot analyses (**Figures 3D, E**; **Supplementary Figure 9D**). We performed CCK-8 assays to explore the roles of FAM72A in the proliferation of the HCC cell lines (Hep3B and HepG2). FAM72A downregulation was closely related to the poor proliferation ability of HCC cells (**Figures 3F, G**). Our study indicated that FAM72A might be applied as a marker for HCC proliferation.

### FAM72A is associated with enhancing the stemness of HCC

**Figure 4:**
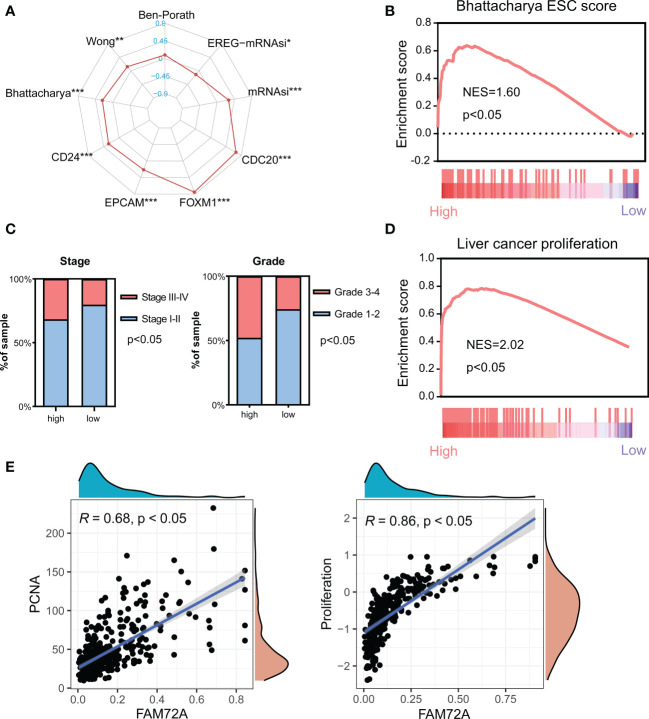
Analysis of the effect of FAM72A on stemness in HCC. **(A)** Radar chart evaluating the relationship between FAM72A expression and the stemness signatures. Statistical analyses were performed using the Spearman correlation test. **(B)** The significant enrichment of high FAM72A expression and the Bhattacharya signature analyzed using GSEA. **(C)** Comparison of the frequency of stages and grades between the high and low FAM72A expression groups. Statistical analyses were performed using the Chi-square test. **(D)** Significant enrichment of high FAM72A expression and the proliferation signature. **(E)** Correlations between FAM72A mRNA expression and both PCNA mRNA levels and proliferation scores. Statistical analyses were performed using the Spearman correlation test. HCC, hepatocellular carcinoma; GSEA, gene set enrichment analysis. *p < 0.05, **p < 0.01, and ***p < 0.001.

Tumor stem cells have an unlimited capacity for self-renewal and division, facilitating tumorigenesis and progression. In our study, FAM72A was closely related to the markers and scores of stem cells (**Figure 4A**). High FAM72A expression was confirmed to be significantly enriched in the Bhattacharya ESC signature (**Figure 4B**). Moreover, the high expression of FAM72A was associated with a significantly larger proportion of tumors with higher stages and grades (**Figure 4C**). A high proliferation capacity is a characteristic phenotype of stem cells. High FAM72A expression was confirmed to be significantly enriched in the signature of “liver cancer proliferation” (**Figure 4D**). A subsequent correlation analysis indicated that FAM72A expression was positively correlated with PCNA expression (R = 0.68, p < 0.05) and proliferation scores (R = 0.86, p < 0.05) (**Figure 4E**). Western blot analysis indicated that PCNA expression in HepG2/Hep3B cells transfected with siFAM72A was obviously inhibited (**Supplementary Figures 9E, F**). Thus, high FAM72A expression tends to result in a dedifferentiated phenotype.

### FAM72A is associated with cancer-promoting mutation profiles in HCC

**Figure 5:**
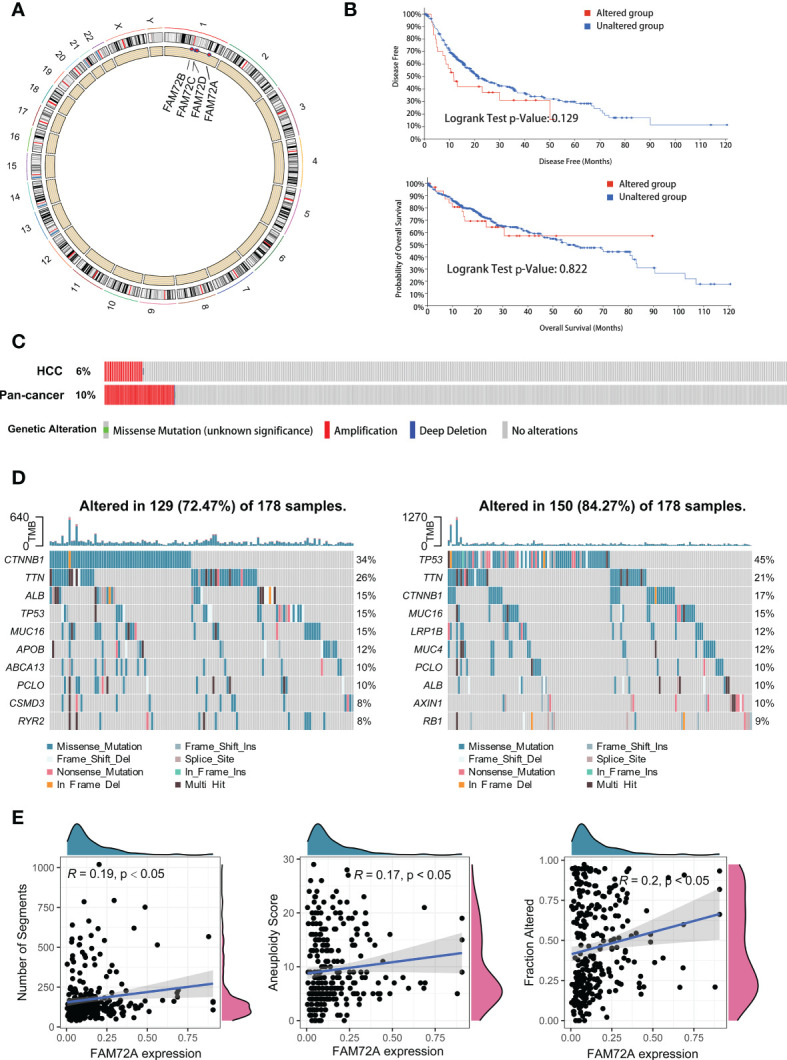
Landscape of genomic alterations and mutation profiles in HCC. **(A)** Chromosomal localization and genetic alterations in FAM72A family genes. **(B)** The cBioPortal database provides the difference in survival, regardless of the presence of FAM72A alterations. Statistical analyses were performed using the log-rank test. **(C)** The cBioPortal database provides the genomic alterations in FAM72A in HCC and pancancer. **(D)** Changes in the somatic mutation frequency between the high (right panel) and low (left panel) FAM72A expression groups. **(E)** Correlation between FAM72A mRNA expression and distinct mutation indices (number of segments, aneuploidy score, and fraction altered). Statistical analyses were performed using the Spearman correlation test.

Genomic variation and gene mutation are important factors related to poor outcomes and the mutation of tumor suppressor genes. Studies have shown that patients with high expression levels of FAM72A are more susceptible to mutations, with possible effects on tumor development. Therefore, we investigated the alterations in FAM72A and their effects on somatic mutations. According to the GRCh38 file, FAM72A is located on chromosome 1, and amplification of this chromosome is the main genetic alteration (**Figure 5A**). We determined the relationship between genetic alterations and survival using the cBioPortal platform. FAM72A gene amplification did not affect the prognosis of patients with HCC (**Figure 5B**). Of HCC patients, FAM72A was amplified in a lower proportion of tissues compared with pan-cancer patients (**Figure 5C**). HCC samples were divided into high and low expression groups according to the median expression of the FAM72A gene. In the low expression group, the frequency of mutations in the pro-oncogene CTNNB1 was significantly higher, while a high frequency of TP53 mutations was verified in the high expression group (**Figure 5D**). Furthermore, FAM72A expression exhibited positive correlations with the mutation scores, such as the number of segments, aneuploidy score and fraction altered (**Figure 5E**).

### The mTOR axis is identified as a target of FAM72A in HCC

**Figure 6:**
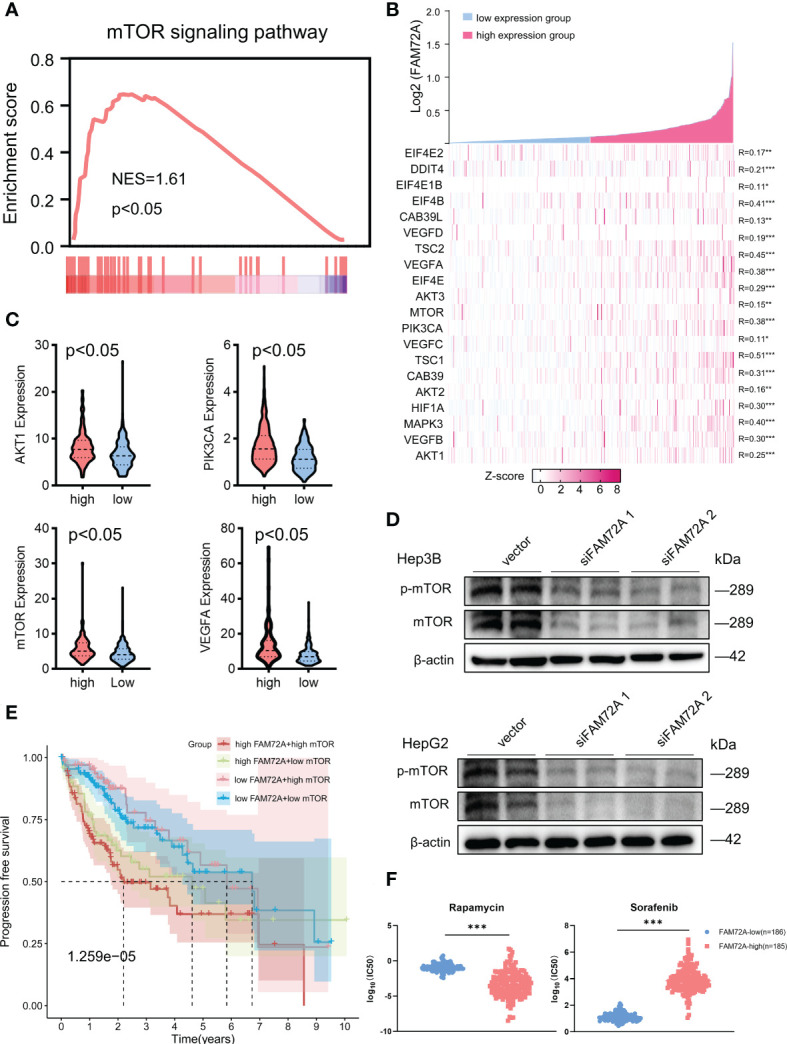
Analysis of the potential relationship between FAM72A and the mTOR axis in HCC. **(A)** Significant enrichment of high FAM72A expression and mTOR signaling pathways. **(B)** Coexpression heatmap of FAM72A and key genes in the mTOR signaling pathway. Statistical analyses were performed using the Spearman correlation test. **(C)** Gene expression levels (AKT1, PIK3CA, mTOR, and VEGFA) in the groups with high and low FAM72A expression. Statistical analyses were performed using the Wilcoxon test. **(D)** Western blot analysis of the relationship between FAM72A and mTOR protein levels in Hep3B and HepG2 cell lines after FAM72A was knocked down. Experiments were repeated three times. **(E)** Survival analysis of individuals with different expression levels of mTOR and FAM72A. Statistical analyses were performed using the log-rank test. **(F)** Differences in drug sensitivity between the high and low FAM72A expression groups. Statistical analyses were performed using the log-rank test. NES, normalized enrichment score; HCC, hepatocellular carcinoma; IC50, half-maximal inhibitory concentration. *p < 0.05, **p < 0.01, and ***p < 0.001.

We performed GSEA of both the high and low expression groups to further elucidate the underlying molecular mechanisms of FAM72A in HCC. GSEA of the KEGG gene sets indicated that the mTOR pathway ranked high and was significantly enriched in the high FAM72A expression group (**Figure 6A**). The top 10 enriched signaling pathways are shown in **Supplementary Table 1**. We further investigated the correlation between the expression of FAM72A and critical genes in the mTOR pathway, and all of these genes had a significant positive correlation with FAM72A (**Figure 6B**). Similarly, AKT1, PIK3CA, mTOR, and VEGFA expression were significantly elevated in the high FAM72A expression group (**Figure 6C**). Through experimental verification, we also detected an equally significant decrease in mTOR expression when FAM72A was knocked down after siRNA transfection in Hep3B and HepG2 cells (**Figure 6D**). A subsequent survival analysis revealed that both FAM72A and mTOR contributed to shorter clinical survival. Patients with high expression of both FAM72A and mTOR had the worst prognosis (**Figure 6E**). Sorafenib (a tyrosine kinase inhibitor) and rapamycin (an mTOR inhibitor) are common therapeutic agents for patients with unresectable HCC. We also explored the effect of FAM72A expression levels on the sensitivity of patients to these drugs. HCC patients with high FAM72A expression levels were presumed to be sensitive to rapamycin but not to sorafenib. High FAM72A expression may be one of the targets mediating sorafenib resistance (**Figure 6F**). Furthermore, by performing CCK-8 assays, we also found that FAM72A overexpression promoted cell proliferation and reduced the inhibitory effect of sorafenib on HepG2 and Hep3B cell proliferation compared with the vector control. However, rapamycin markedly enhanced the inhibitory effect of sorafenib on HepG2 and Hep3B cell proliferation (**Supplementary Figure 10A, B**).

### FAM72A might be associated with the TME, according to GO and KEGG enrichment analyses

**Figure 7:**
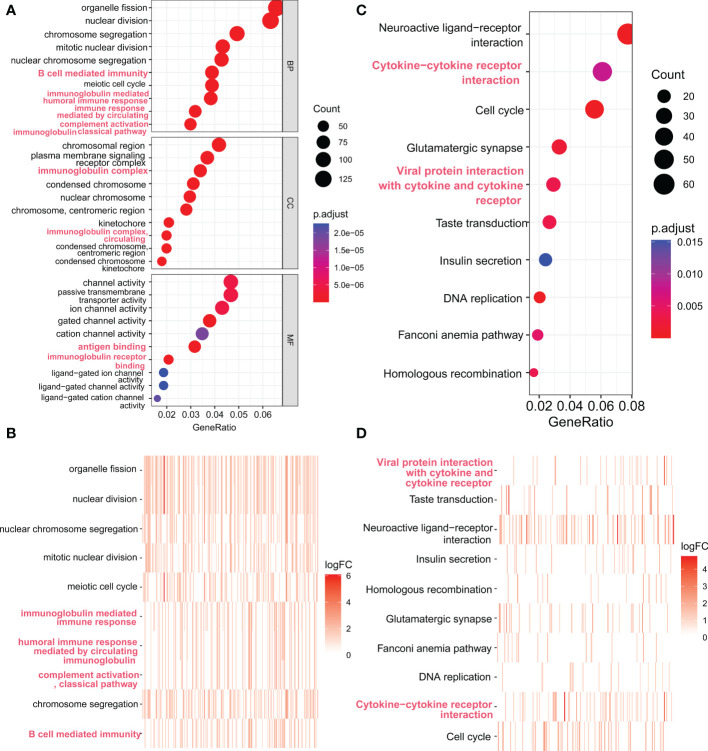
Functional enrichment analysis of genes expressed at significantly higher levels in the high FAM72A expression group. **(A, B)** Scatter plot and heatmap of the top 10 enriched GO terms sorted by GeneRatio. **(C, D)** Scatter plot and heatmap of the top 10 enriched KEGG pathways sorted by GeneRatio.

We further investigated FAM72A-related genes in HCC by performing enrichment analyses. Differentially expressed genes were identified between the high and low FAM72A expression groups in TCGA-LIHC cohort. We selected 3619 genes with logFC > 1 and p < 0.05 for subsequent enrichment analyses. The top 10 enriched pathways are shown in **Figure 7** and revealed that FAM72A-related gene sets are significantly enriched in immune-related pathways, such as B cell-mediated immunity, immune response, immunoglobulin complex circulating, antigen binding, and cytokine−cytokine receptor interaction. These results implied that FAM72A appears to be associated with immune regulation.

### High FAM72A expression correlates with activated infiltrating immune cell populations and sensitivity to immunotherapy

**Figure 8:**
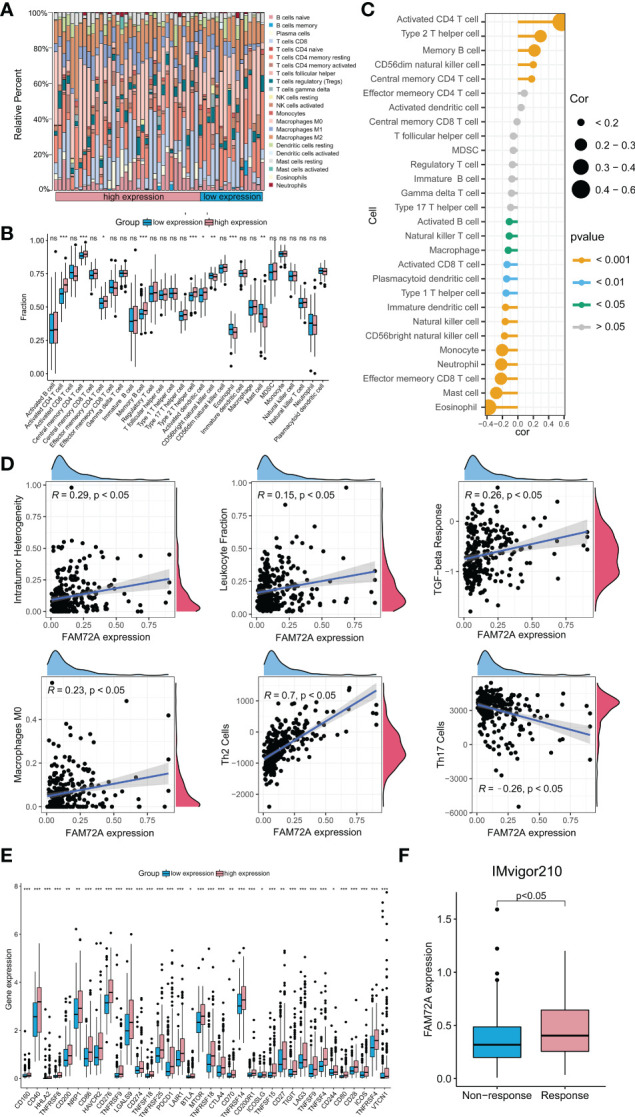
Immune-related analysis of FAM72A in HCC. **(A)** The difference in the abundance of 29 immune cell types between the high and low FAM72A expression groups. **(B)** Correlation between FAM72A mRNA expression and immune cell infiltration. Statistical analyses were performed using the Wilcoxon test. **(C)** The proportions of 22 tumor-infiltrating immune cell types in the high and low FAM72A expression groups were evaluated using the CIBERSOFT algorithm. Statistical analyses were performed using the Spearman correlation test. **(D)** Correlation between FAM72A mRNA expression and immune scores. Statistical analyses were performed using the Spearman correlation test. **(E)** The differences in expression of 36 immune checkpoints between the high and low FAM72A expression groups. Statistical analyses were performed using the Wilcoxon test. **(F)** The relationship between FAM72A expression and the efficiency of immunotherapy in the IMvigor210 cohort. Statistical analyses were performed using the Wilcoxon test. HCC, hepatocellular carcinoma. *p < 0.05, **p < 0.01, and ***p < 0.001.

We detected the relationship between FAM72A overexpression and immune cell infiltration in the HCC microenvironment to confirm the crucial regulatory role of FAM72A in the differentiation and activation of tumor immunity. Using the CIBERSOFT algorithm, the composition of 22 types of immune cells was computed in subgroups stratified by different FAM72A expression levels. Compared with the FAM72A-low group, the proportions of activated CD4 memory T cells, plasma cells, and macrophages seemed different in the FAM72A-high group (**Figure 8A**). Furthermore, we scored the samples based on 28 infiltrating cell types between the FAM72A-low and FAM72A-high groups using the ssGSEA method. The high expression group contained a significantly higher proportion of activated CD4^+^ T cells, memory CD4^+^ T cells, memory B cells, Th2 helper T cells, and activated dendritic cells. In contrast, fewer infiltrating eosinophils and mast cells were detected. Meanwhile, the correlation analysis revealed that FAM72A expression significantly correlated with immune cell subpopulations, especially activated CD4^+^ T cells (**Figure 8B**). In addition, FAM72A expression was positively correlated with the content of type 2 T helper cells but negatively correlated with that of type 1 T helper cells (**Figure 8C**). Furthermore, because transcriptome-based immune-related scores, Th17 cells, and macrophage subtypes are closely related to the progression of HCC, we investigated their correlations with the FAM72A expression profile. Not surprisingly, high FAM72A expression was associated with intratumor heterogeneity (R = 0.29; p < 0.05), the leukocyte fraction (R = 0.15; p < 0.05), TGF-β response (R = 0.26; p < 0.05) and the abundance of M0 macrophages (R = 0.23; p < 0.05), Th2 cells (R = 0.7; p < 0.05) and Th17 cells (R = -0.26; p < 0.05) (**Figure 8D**). Immunotherapy efficacy depends not only on the presence of sufficient immune cell infiltration in the microenvironment but also on the high expression of immune checkpoints. Therefore, we further explored the differences in immune checkpoint expression between the groups with high and low FAM72A expression. A substantial difference in the expression of all 34 immune checkpoints was observed (**Figure 8E**). We further evaluated the relationship between the expression of FAM72A and the effect of immunotherapy using the IMvigor210 database (**Figure 8F**). Patients with higher FAM72A expression were more sensitive to immunotherapy. These results supported the findings that FAM72A might function as an important potential target for immunotherapy in HCC. Given the potential relationship between mTOR and FAM72A, we also explored the association between mTOR and immunity. The results were obtained from the TIME2.0 database, and, similar to FAM72A, mTOR expression was positively correlated with the levels of infiltration of multiple immune cell types, such as CD4^+^ T cells and helper type T cells (**Supplementary Figure 11A**). In addition, mTOR also showed a positive association with immune checkpoint expression (**Supplementary Figure 11B**). By analyzing the IMvigor210 database, the expression levels of mTOR were used to assess the efficacy of immunotherapy (**Supplementary Figure 11C**).

## Discussion

FAM72A is reported to be a neuronal protein that is rarely expressed in other tissues under physiological conditions (28). Previous studies have shown that FAM72A is associated with the occurrence, development, and prognosis of various malignant tumors in nonneural tissues, such as colon, breast and lung cancers (9, 18). According to a recent study, increased levels of FAM72A might stimulate cancer initiation and development and induce treatment resistance (14). However, the expression and function of FAM72A in HCC remain unclear. For the first time, we analyzed FAM72A expression, diagnostic and prognostic values, correlations with stemness and mutations, potential downstream pathways, and associations with immune cell infiltration and immune checkpoints in HCC using multiple databases.

First, we investigated FAM72 gene expression across cancers and identified that high expression of FAM72 genes might contribute to a poor patient prognosis and regulate immune cell infiltration in many cancers. The results suggested that FAM72 might be a useful prognostic predictive biomarker and a potential therapeutic target. The clinical significance of FAM72 family members in HCC was further explored comprehensively. The expression analysis revealed that FAM72 members were significantly overexpressed in HCC tissues compared to normal tissues. This result is consistent with previous pancancer results. One study reported that FAM72A expression was significantly increased in human lung cancer, and FAM72A is a potential molecular marker for a poor prognosis (18). Another study suggested that high FAM72 expression is a prognostic risk factor for glioblastoma multiforme (17). Therefore, we evaluated the diagnostic and prognostic values of FAM72 family members for HCC. Cox regression analysis showed that FAM72A and B are potential independent prognostic biomarkers. A subsequent ROC curve analysis showed that FAM72A has a strong power in differentiating HCC patients from healthy subjects (AUC > 0.9). The stratified prognostic analysis suggested that FAM72A is a prognostic biomarker for patients stratified by sex, age and early-stage HCC. We also identified that FAM72A was significantly less frequently mutated in HCC than in other cancers. FAM72A is relatively conserved in HCC. These results implied that FAM72A might represent a potentially useful prognostic biomarker for HCC patients.

Previous studies have shown that high FAM72A expression maintains cell viability for a long time, promotes stem cell renewal and proliferation, and increases stemness levels (24), consistent with the results from our study. Our research also revealed the relationship between high expression levels of FAM72A and stemness scores in HCC. Moreover, mutations in EGFR, TP53, PTEN, NF1, SPTA1, PIK3CA or SCN9A, MXRA5, ADAM29, KDR, PIK3C2G, and LRP1B were associated with high FAM72 expression and postulated to lead to cell cycle activation, cell transformation, and cell proliferation (6). The average frequency of TP53 mutations in HCC was 30% (29). In the present study, we found that TP53 mutations were present in nearly half of the patients in the group with high FAM72A expression. As one of the most common oncogenes, mutations in TP53 are associated with hepatocellular carcinoma differentiation, vascular invasion, serum alpha-fetoprotein (AFP) levels, and tumor stage (29, 30). Meanwhile, accumulated LRPPRC and MDR1 promote tumor resistance when TP53 is mutated (31). Therefore, specific management of HCC patients with high expression of FAM72A is essential.

The mTOR signaling pathway, a central regulator of various cellular processes, integrates intracellular and extracellular signals, including those involved in cell growth, proliferation, metabolism, and survival (32). Oncogenic activation of mTOR signaling is commonly observed in HCC samples. A clear correlation between activation of the PI3K/AKT/mTOR pathway and poor clinical outcomes in HCC patients has been documented (33). This evidence provides a rationale for further studies examining the possible use of mTOR inhibitors to treat HCC. In our study, high FAM72A expression was significantly associated with activation of the mTOR pathway. Patients with high expression of both FAM72A and mTOR had the worst prognosis. Through drug sensitivity studies, we found that patients with high FAM72A expression were more sensitive to rapamycin, while those treated with sorafenib mainly developed resistance, consistent with previous studies. Chen et al. (32) reported significantly increased activation of the mTOR pathway in the acquired sorafenib resistance clonal phenotype. In addition, targeting the mTOR signaling pathway increases sorafenib inhibition of HCC cells (34–36). A combination sorafenib and rapamycin might be preferable for patients with high FAM72A expression.

CD4^+^ T cells are presumed to be involved in antitumor immune responses by secreting cytokines such as IL-2, IL-10, TGF-β, and IL-35 (37). CD4^+^ T cells inhibit CD8^+^ T cells, as evidenced by the suppressed proliferation and activation, diminished cytotoxicity of CD8^+^ T cells, and reduced production of granzyme A/B (38). By promoting antigen-specific immune escape, tumors tend to be more malignant. Our study showed that high FAM72A expression is closely associated with a high abundance of CD4^+^ T cells. Th2 cells, an independent risk factor for the proliferation and progression of HCC, are associated with immunosuppression and cancer cell migration (39). Th17 cells improve the survival rate of HCC patients by secreting IL-17 (40). The profile of Th2/Th17 cytokines in HCC is unbalanced. The cytokine profile shifted to a Th17 phenotype, leading to the induction of an antitumor effect. Our study revealed that high FAM72A expression levels positively correlated with Th2 proportions but negatively correlated with Th17 proportions. In addition, we compared the differences in immunotherapy-related indicators between patients in the high and low FAM72A expression groups and found that the expression of multiple immune checkpoint molecules (such as PD-L1, CTLA4, and LAG3) was elevated in the high expression group. Patients in the high expression group had a better immunotherapy response. These results indirectly suggest that FAM72A may play a key role in predicting the effect of immunotherapy. In addition, we found that mTOR expression was associated with the expression of some immune checkpoint molecules. Therefore, we speculate that FAM72A might regulate the immune response through the mTOR pathway.

To the best of our knowledge, this study is the first to systematically investigate the value of FAM72A in HCC. Among FAM72 family genes, FAM72A was indicated to be a promising target for the diagnosis, prognostic prediction, and therapeutic intervention of HCC. Naturally, some limitations of our research should be addressed. First, the prognostic value of FAM72A also must be validated in a real clinical cohort. The database employed for the study lacks posttranslational modifications and therefore cannot provide a comprehensive clarification of how these modifications may affect the function of FAM72A. Second, the carcinogenic mechanism of FAM72A warrants intensive study. Third, FAM72A upregulation only indirectly provides evidence for changes in the TME but not direct evidence. The relationship between FAM72A and the TME is not well defined. In addition, the mechanism by which high FAM72A expression may cause sorafenib resistance remains to be further explored.

## Conclusions

In conclusion, FAM72A represents a potential molecular marker to diagnose and predict the prognosis of HCC patients. FAM72A might be a critical protein activating HCC stemness, oncogenic mutations, and immune escape. Our study extends previous findings by identifying the FAM72A/mTOR axis as a potential therapeutic target for HCC, which should be confirmed in the HCC population.

## Data availability statement

The datasets presented in this study can be found in online repositories. The names of the repository/repositories and accession number(s) can be found in the article/**Supplementary Material**.

## Ethics statement

The studies involving human participants were reviewed and approved by the Research Ethics Committee of The Third Affiliated Hospital of Sun Yat‐Sen University. The patients/participants provided their written informed consent to participate in this study. The animal study was reviewed and approved by the Institutional Animal Care and Use Committee at the Third Affiliated Hospital of Sun Yat-Sen University.

## Author contributions

All authors contributed to the study conception and design. QZ and LC contributed equally to this study. QZ and LC designed and wrote the manuscript. QZ and YC screened the literature and collected the data. QZ and LY were responsible for creating the pictures and performing statistical analyses. QZ and HZ polished the article. YG critically revised the manuscript. All authors commented on previous versions of the manuscript. All authors read and approved the final manuscript.

## Funding

This work was sponsored by a grant from the National Natural Science Foundation of China (82170604).

## Acknowledgments

We acknowledge the TCGA, ICGC, IMvigor210, cBioportal, GeneMANIA, and TIMER databases for providing their platforms and the contributors for uploading their meaningful datasets.

## Conflict of interest

The authors declare that the research was conducted in the absence of any commercial or financial relationships that could be construed as a potential conflict of interest.

## Publisher’s note

All claims expressed in this article are solely those of the authors and do not necessarily represent those of their affiliated organizations, or those of the publisher, the editors and the reviewers. Any product that may be evaluated in this article, or claim that may be made by its manufacturer, is not guaranteed or endorsed by the publisher.
