# Supplementary material for: Integrated systemic analysis of FAM72A to identify its clinical relevance, biological function, and relationship to drug sensitivity in hepatocellular carcinoma
Source: Front Oncol. 2022 Nov 22;12:1046473. doi: 10.3389/fonc.2022.1046473 (PMC9723133; doi:10.3389/fonc.2022.1046473)
Supplement: Supplementary file 1 [file DataSheet_1.docx]

Supplementary Material

## 1.1 Supplementary Figures

**
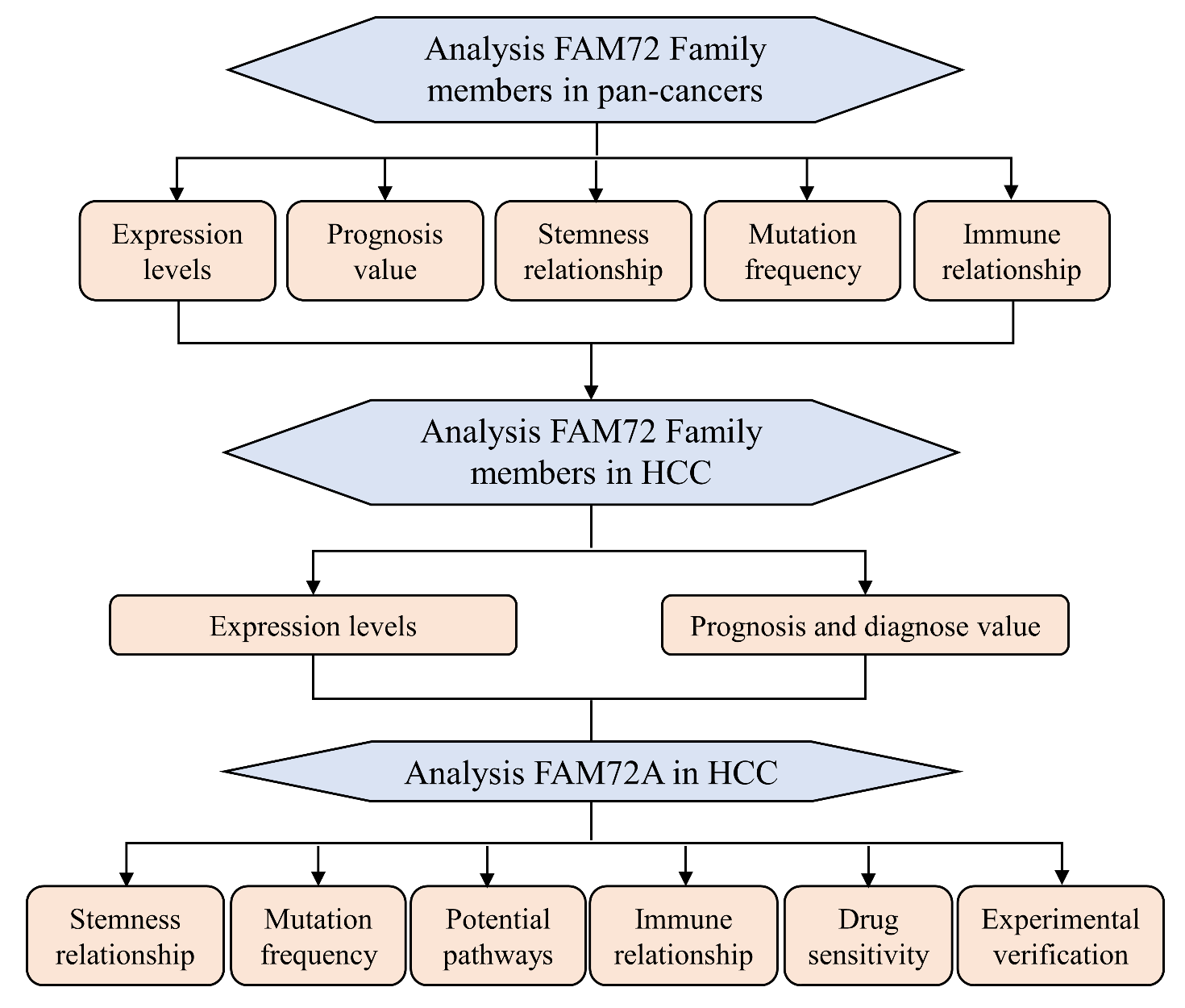
**

**Supplementary Figure 1.** The procedure of the study. HCC, hepatocellular carcinoma

**
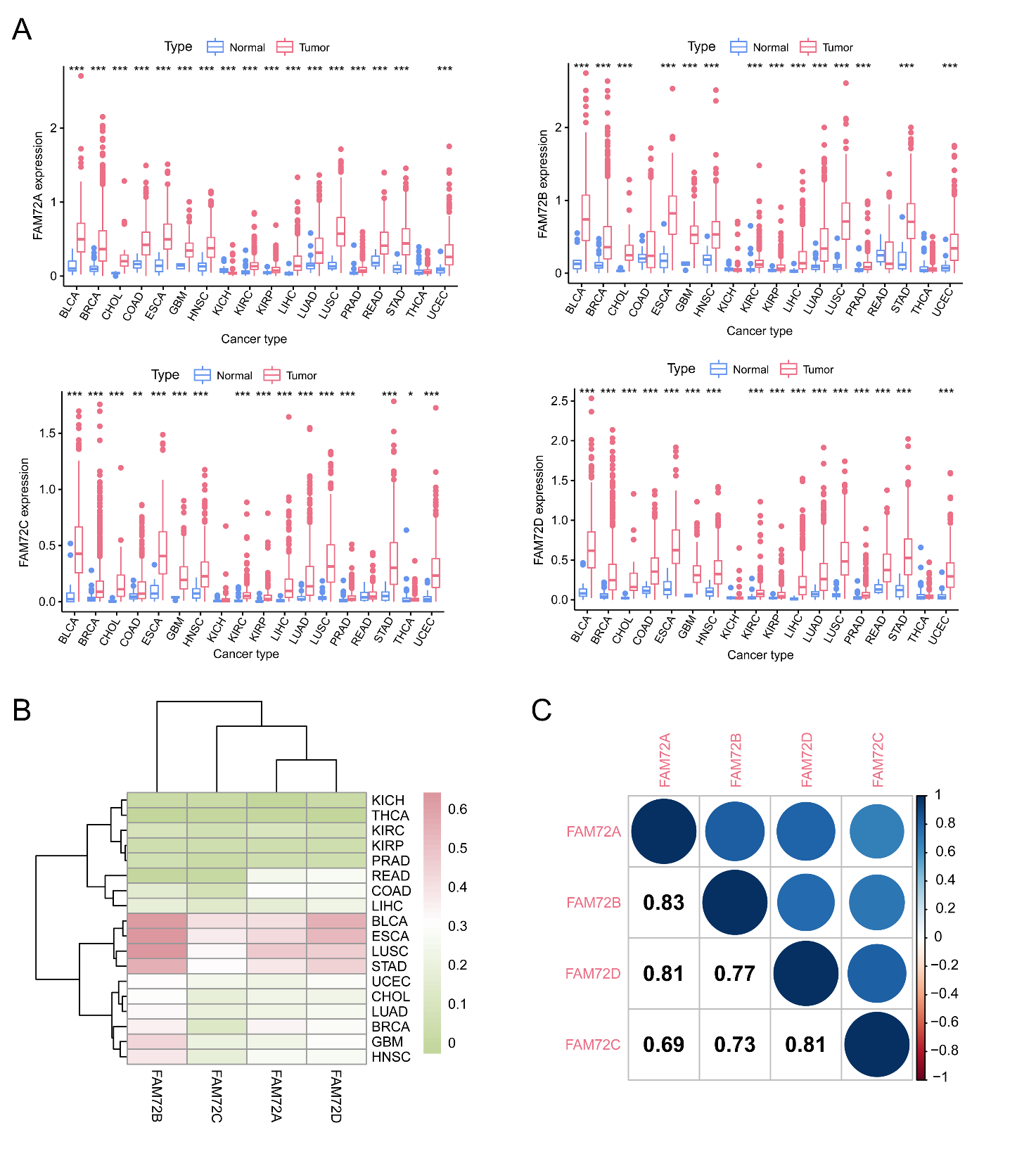
**

**Supplementary Figure 2.** The expression profiles of FAM72 family members in pan-cancer. **(A)** The expression levels of FAM72A family members in 18 types of tumor. Statistical analyses were done using Wilcoxon test. **(B)** The heatmap of the expression profiles. **(C)** The correlation among FAM72A family members’ expression. Statistical analyses were done using Spearman correlation test. BLCA, bladder urothelial carcinoma; BRCA, breast invasive cancer; CHOL, cholangiocarcinoma; COAD, colon adenocarcinoma; ESCA, esophageal carcinoma; GBM, glioblastoma multiforme; HNSC, head and neck squamous cell carcinoma; KICH, kidney chromophobe; KIRC, kidney renal clear cell carcinoma; KIRP, kidney renal papillary cell carcinoma; LIHC, liver hepatocellular carcinoma; LUAD, lung adenocarcinoma; LUSC, lung squamous cell carcinoma; PRAD, prostate adenocarcinoma; READ, rectum adenocarcinoma; STAD, stomach adenocarcinoma; THCA, thyroid Carcinoma; UCEC, uterine Corpus Endometrial Carcinoma. *p < 0.05; ** p < 0.01; *** p< 0.001.


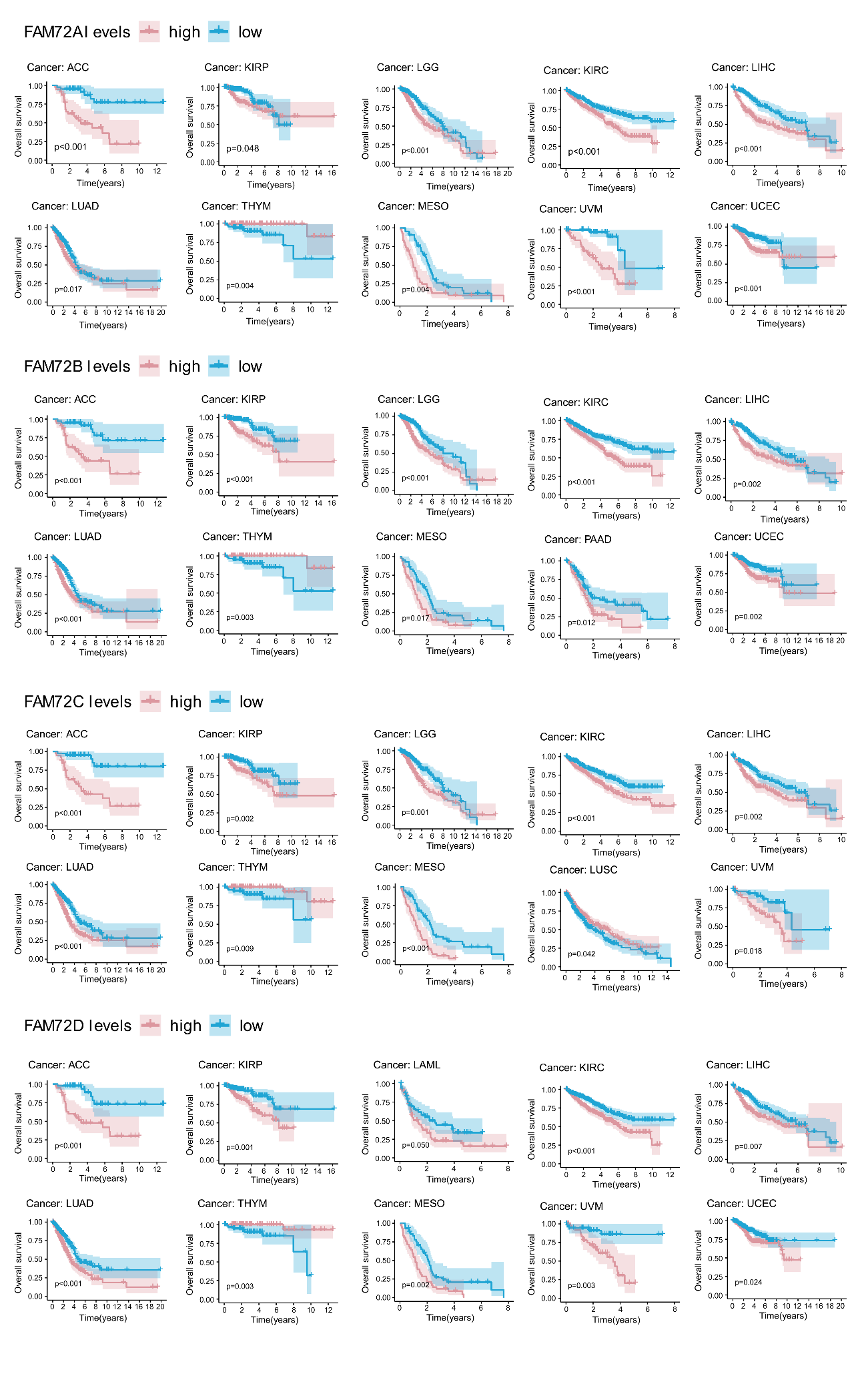


**Supplementary Figure 3.** OS Kaplan-Meier curves for patients with different levels of FAM72 family members. Statistical analyses were done using Log-rank test. The full name of the tumor abbreviation is the same as shown in Supplement Figure 1.


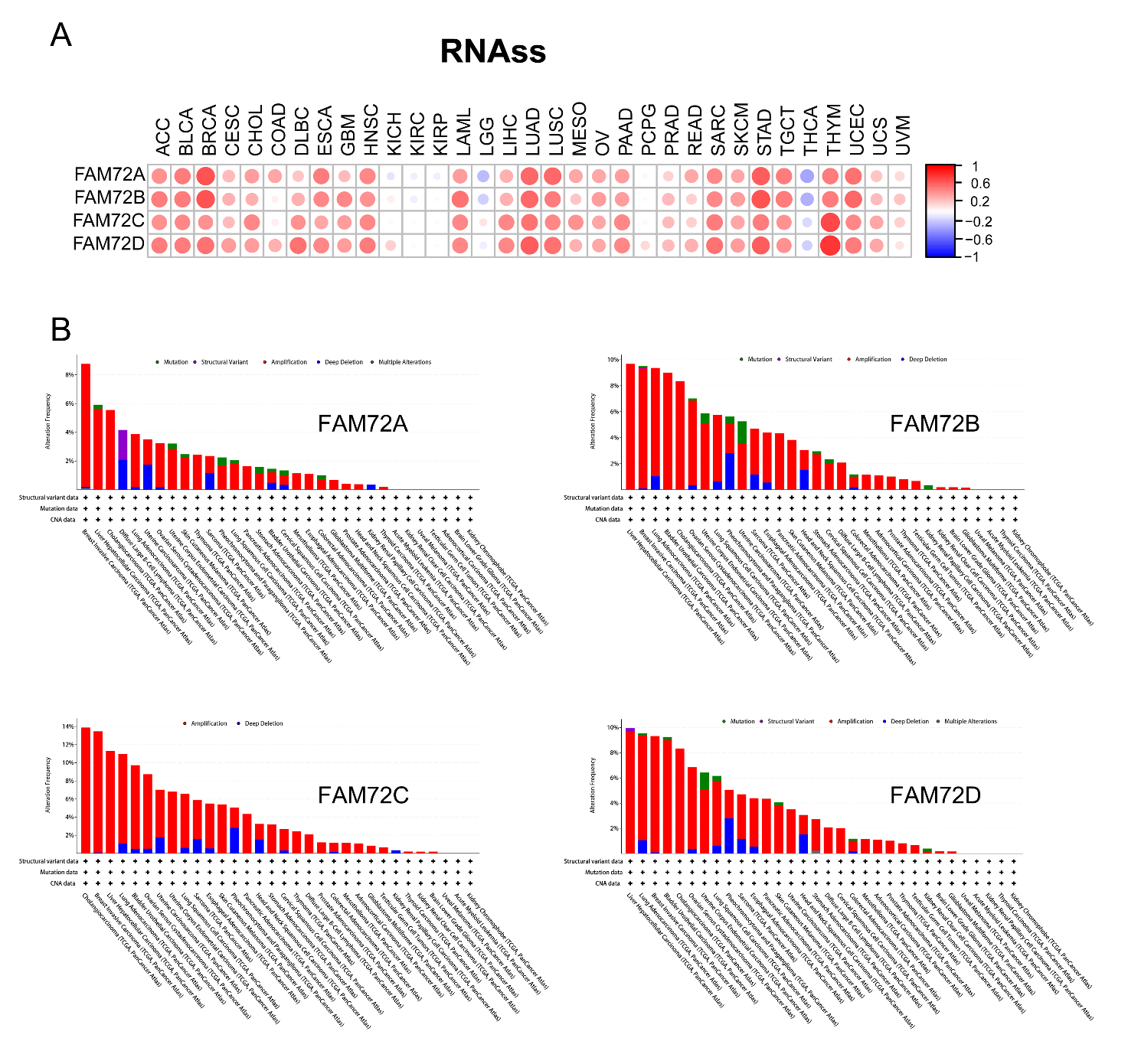


**Supplementary Figure 4.** Stemness and genetic alterations of the FAM72 gene family in different tumors. **(A)** Correlation of FAM72 gene family expression and RNAss. Statistical analyses were done using Spearman correlation test. **(B)** Mutation character based on the Cbioportal database in pan-cancer. The full name of the tumor abbreviation is the same as shown in Supplement Figure 1.
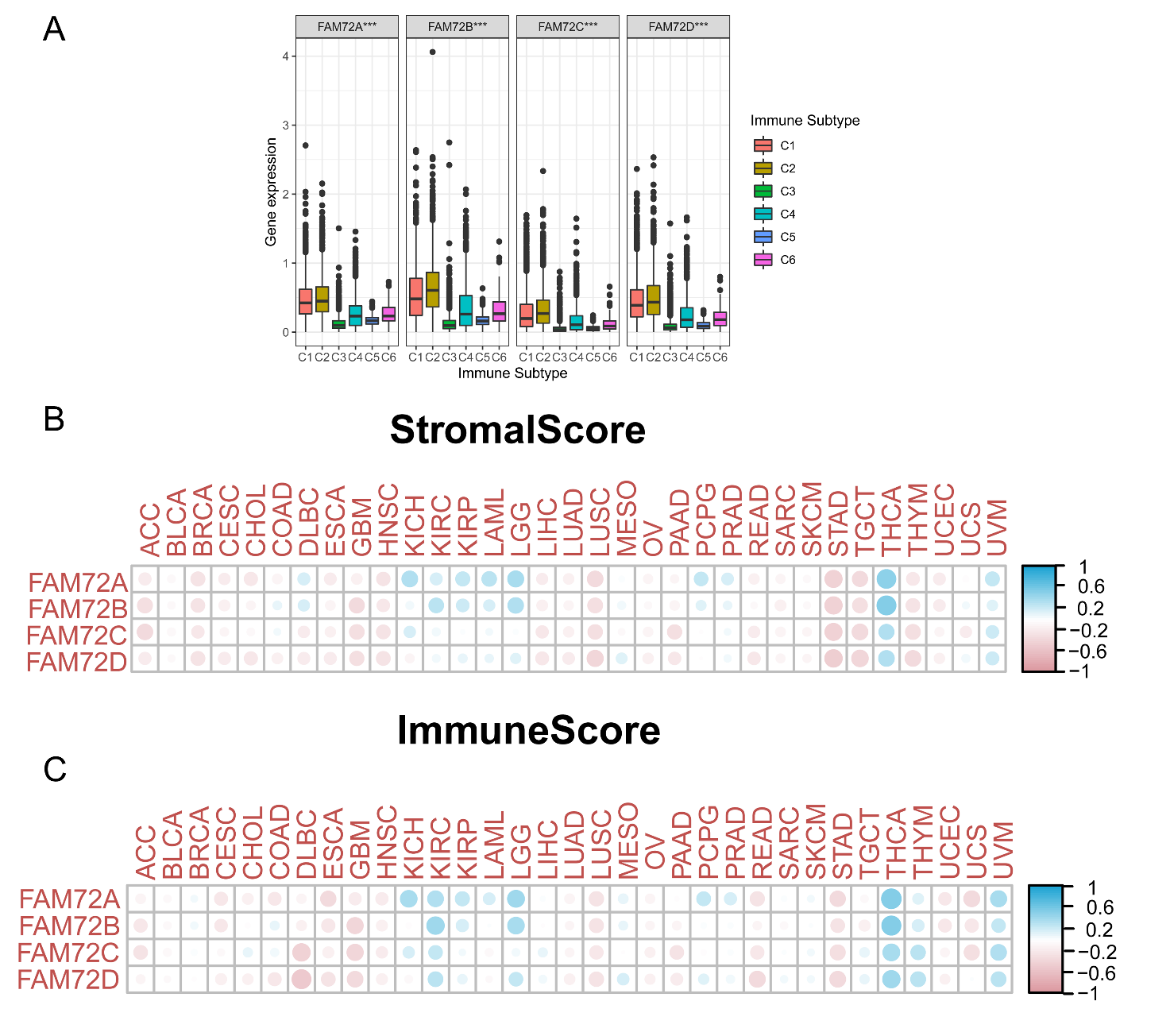


**Supplementary Figure 5.** The association between FAM72 expression and immune cell infiltration. **(A)** Differential expression of FAM72 in different immunophenotypes. Statistical analyses were done using Kruskal-Wallis test. **(B, C)** Correlation matrix plots show the correlation of FAM72 with stromal score and immune score. The size of the points represents the correlation coefficient. The larger the point, the higher the correlation. Statistical analyses were done using Spearman correlation test. The full name of the tumor abbreviation is the same as shown in Supplement Figure 1. C1, wound healing; C2, IFN-γ dominant; C3, inflammatory; C4, lymphocyte depleted; C5, immunologically quiet; C6, TGF-β dominant. *P < 0.05; **P < 0.01; ***P < 0.001.
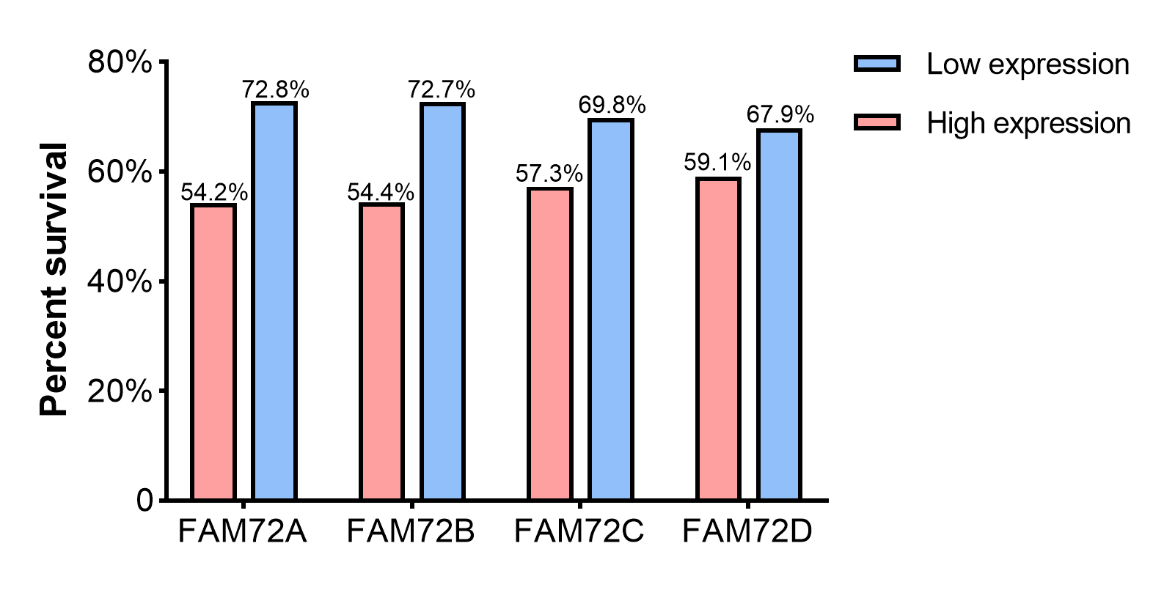


**Supplementary Figure 6.** Five-year overall survival of HCC patients with high and low expression of FAM72 family genes in the TCGA-LIHC dataset. The high and low groups are determined by the median expression value of each gene. HCC, hepatocellular carcinoma
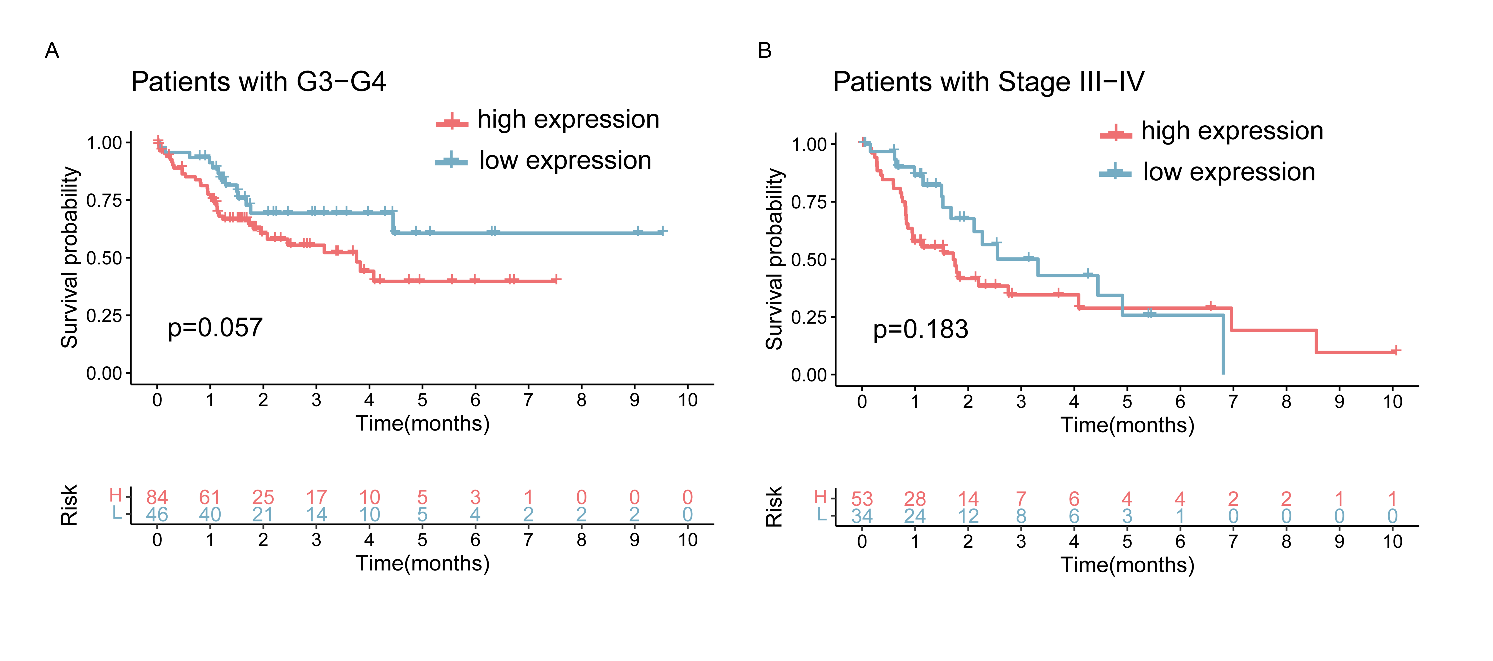
**Supplementary Figure 7.** Predictive survival ability of different expression levels of FAM72A for patients with G3-G4 or Stage III-IV.

**
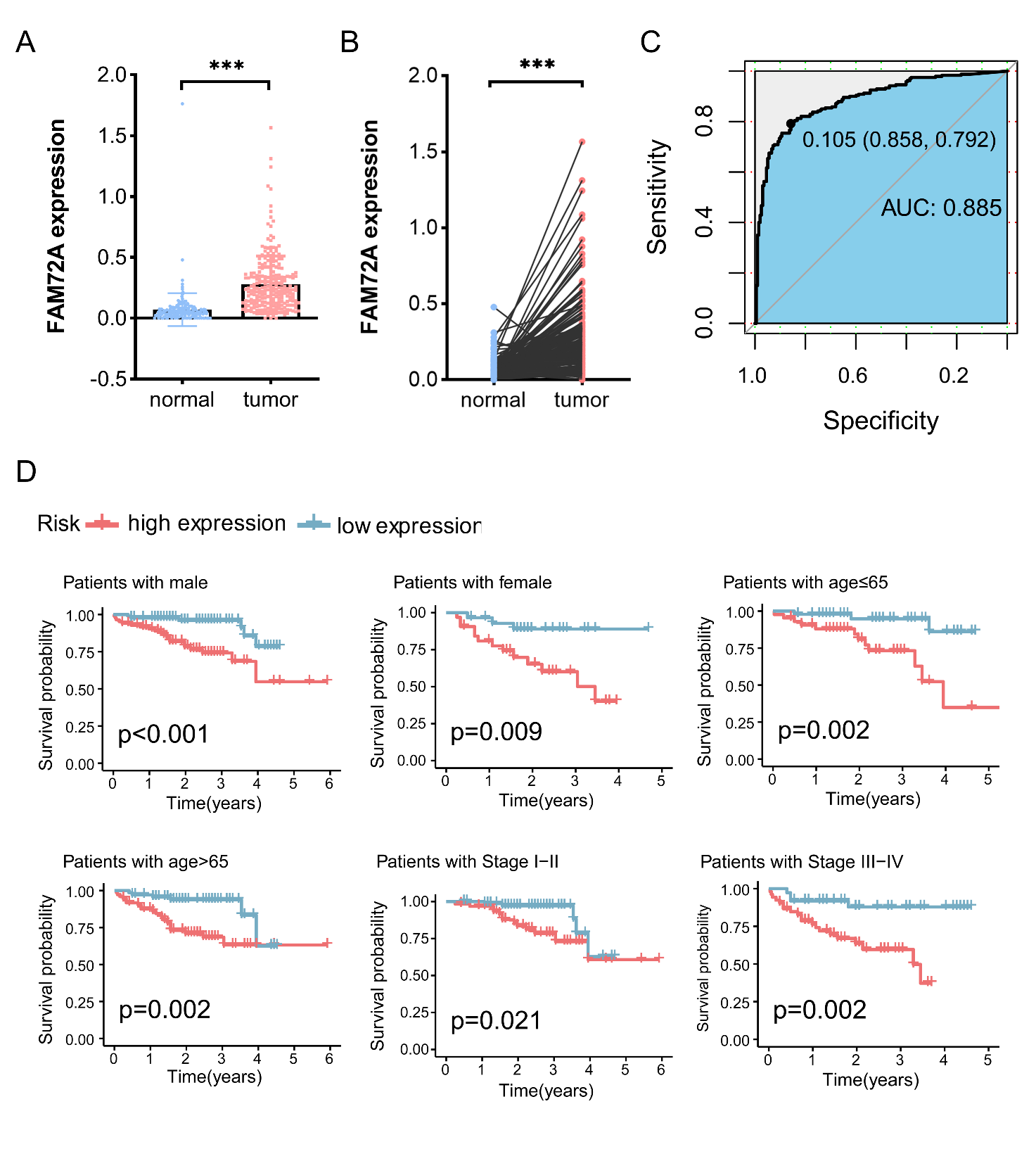
Supplementary Figure 8.** FAM72A analysis in ICGC-LIRI-JP. **(A)** Comparison the expression of FAM72A between normal and tumor groups. Statistical analyses were done using Wilcoxon test. **(B)** Analysis of FAM72A expression in pairs of HCC and adjacent normal tissues. Statistical analyses were done using Student’s t test. **(C)** ROC analysis in diagnosis values of FAM72A. **(D)** Kaplan-Meier survival curves comparing the high and low expression of FAM72A in the different clinicopathological stratifications. Statistical analyses were done using Log-rank test. HCC, hepatocellular carcinoma; ROC, receiver‐operating characteristic. *** p< 0.001.


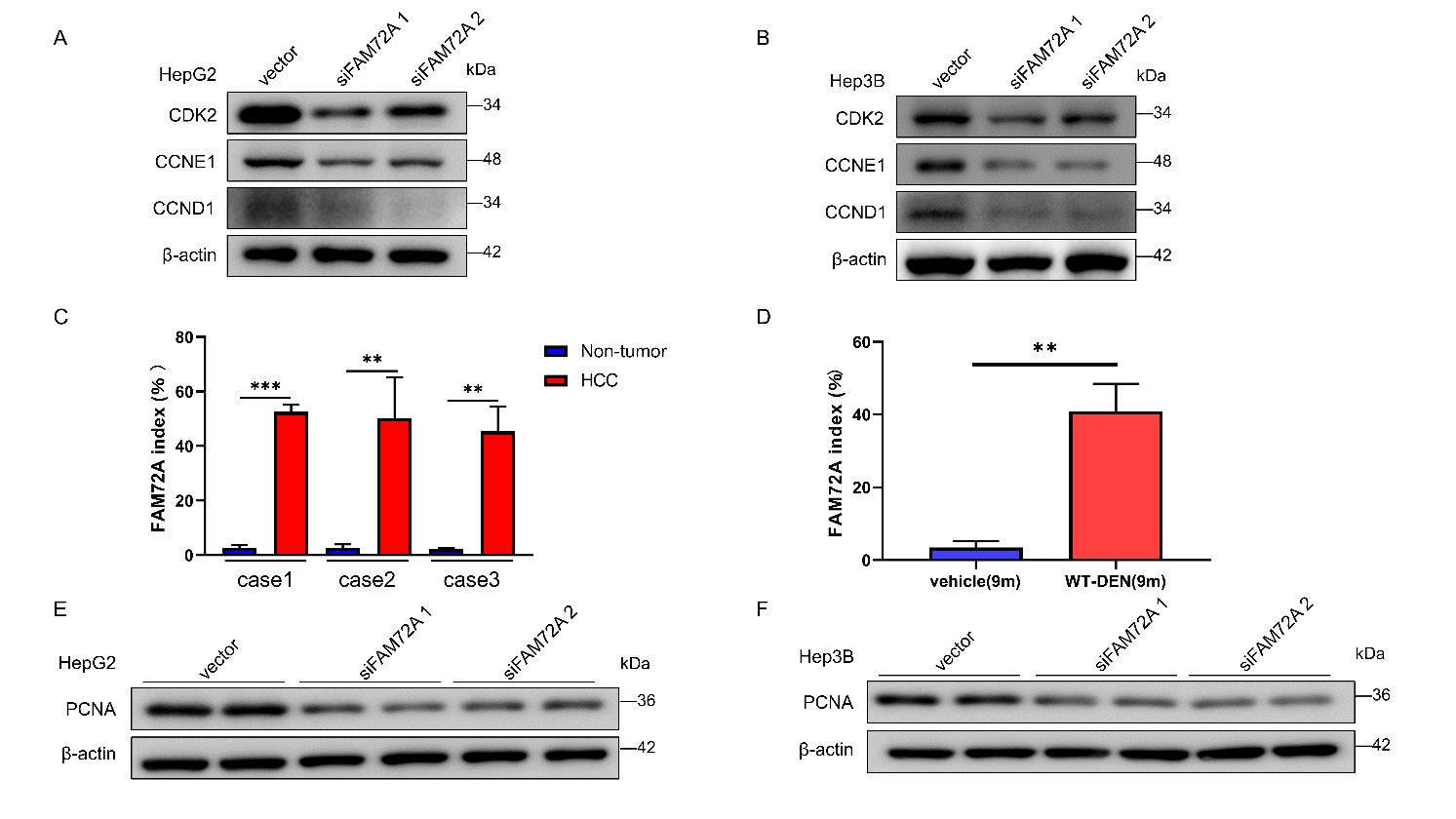
**Supplementary Figure 9.** Experimental validation of FAM72A in HCC. **(A, B)** Western blot analysis of CDK2, CCNE1, CCND1, and CDK2 expression in HepG2/Hep3B cells transfected with vector, siFAM72A1 and siFAM72A2. β-actin was used as the loading control. **(C)** FAM72A staining in human HCC and paracancerous tissues. **(D)** FAM72A staining in WT (vehicle) and WT-DEN mice tissues. **(E, F)** Western blot analysis of PCNA expression in HepG2/Hep3B cells transfected with vector, siFAM72A1 and siFAM72A2. β-actin was used as the loading control. Error bars are means ±SD of triplicate experiments. Statistical analyses were done using Student’s t test. HCC, hepatocellular carcinoma; **p < 0.01; ***p < 0.001.
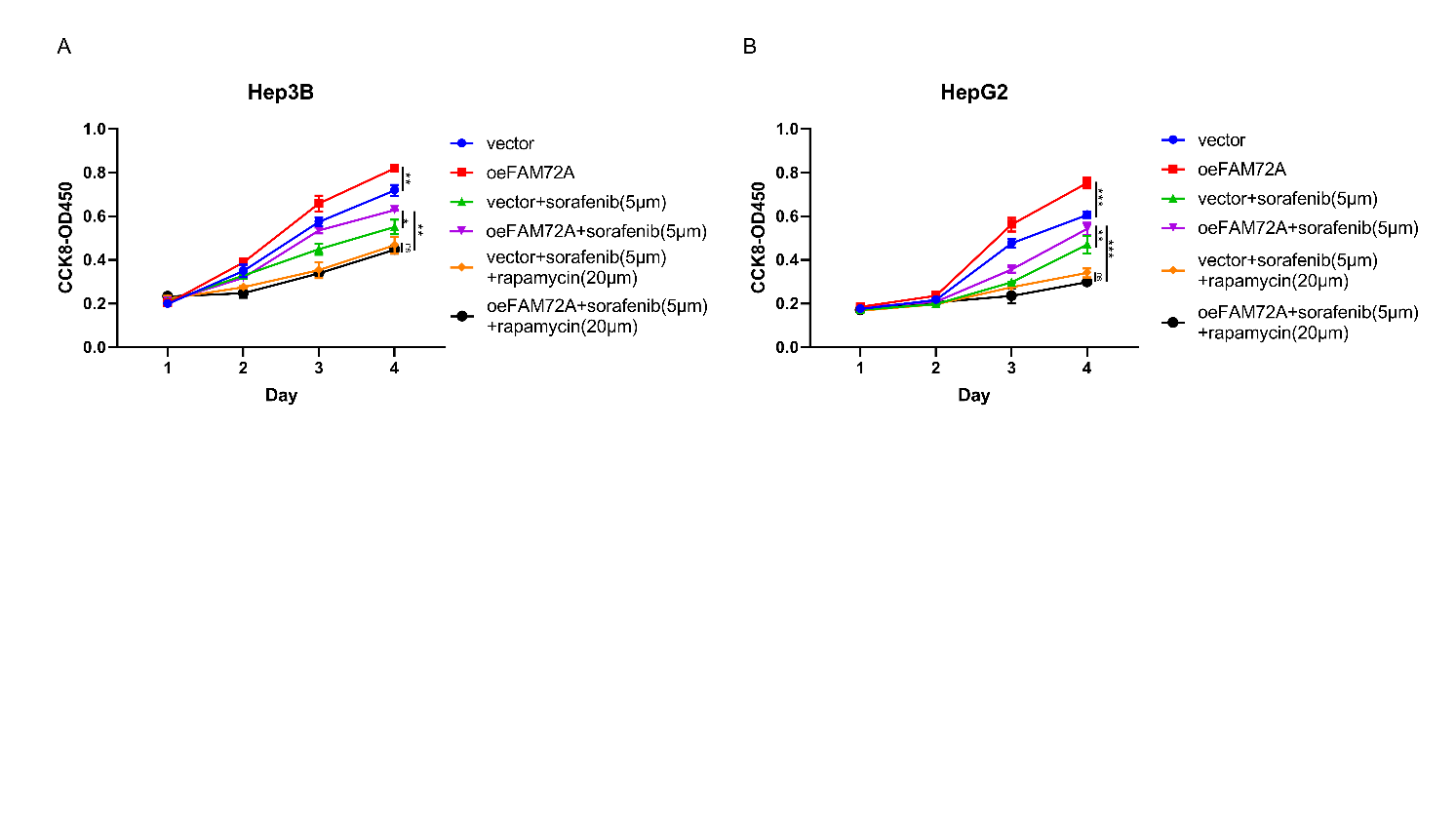


**Supplementary Figure 10.** Effect of cell growth of transfected vector and oeFAM72A Hep3B **(A)**/HepG2 cells **(B)** treated with sorafenib (5μm) and/or Rapamycin (20μm) by CCK-8 assay. Statistical analyses were done using One-way ANOVA. Above experiments were repeated three times. CCK-8, Cell Counting Kit-8. *p < 0.05; **p < 0.01; ***p < 0.001.

**
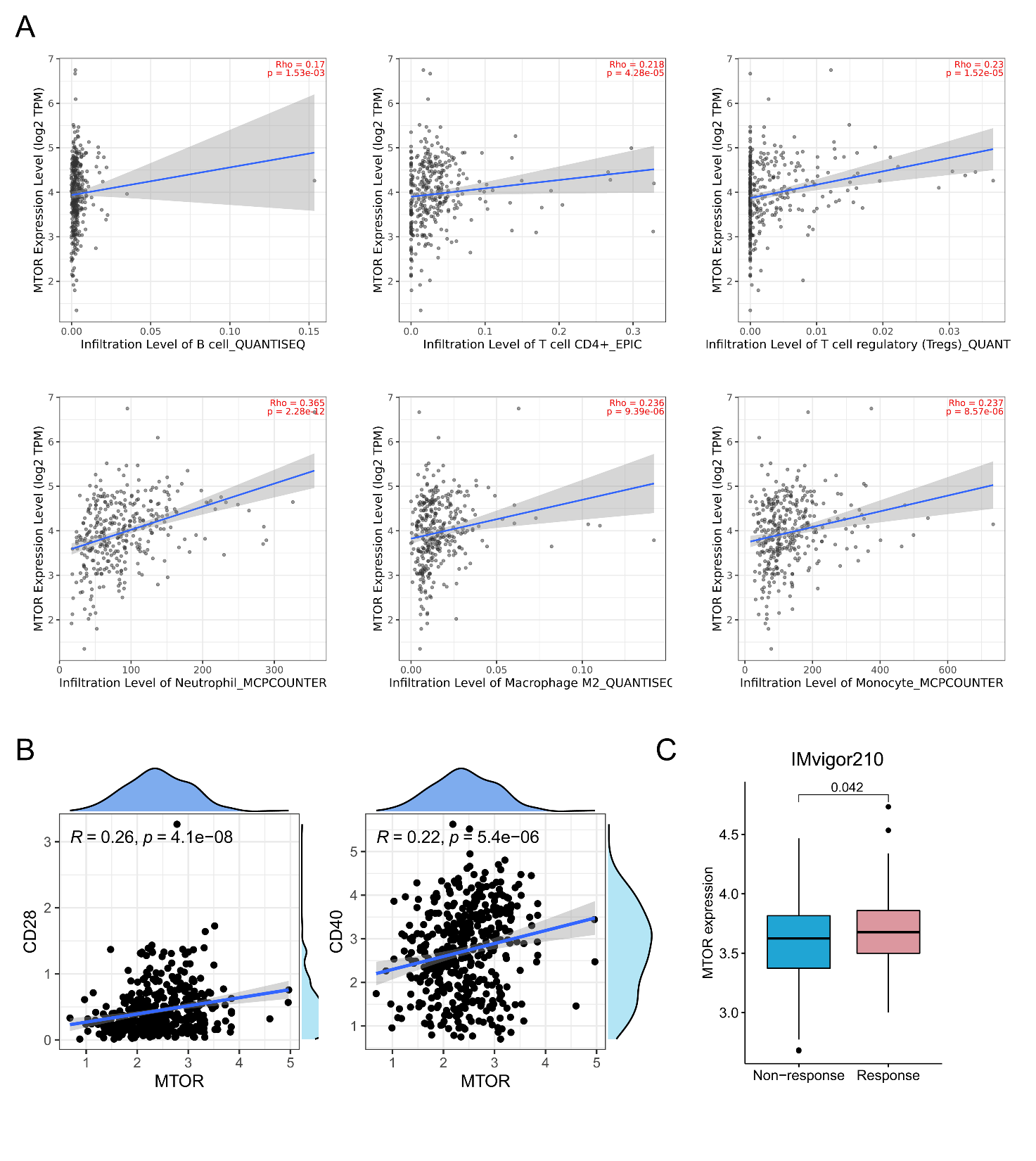
Supplementary Figure 11.** The association between mTOR expression and immune. **(A)** Correlation between the mTOR mRNA expression and immune cells-infiltration based on the TIMER2.0 database. **(B)** Correlation between FAM72A and immune checkpoint genes. Statistical analyses were done using Spearman correlation test. **(C)** The relationship between mTOR expression and efficiency of immunotherapy in the IMvigor210 cohort. Statistical analyses were done using Wilcoxon test.

## 1.2 Supplementary Tables

**Supplementary Table 1.** The gene set enriched in higher FAM72A expression phenotype

| Gene set name | NES | NOM p-val | FDR q-val |
| --- | --- | --- | --- |
| KEGG_NEUROTROPHIN_SIGNALING_PATHWAY | 1.726 | <1×10^-5^ | <1×10^-5^ |
| KEGG_PHOSPHATIDYLINOSITOL_SIGNALING_SYSTEM | 1.641 | <1×10^-5^ | <1×10^-5^ |
| KEGG_MTOR_SIGNALING_PATHWAY | 1.595 | <1×10^-5^ | <1×10^-5^ |
| KEGG_T_CELL_RECEPTOR_SIGNALING_PATHWAY | 1.593 | <1×10^-5^ | <1×10^-5^ |
| KEGG_ERBB_SIGNALING_PATHWAY | 1.567 | <1×10^-5^ | <1×10^-5^ |
| KEGG_RIG_I_LIKE_RECEPTOR_SIGNALING_PATHWAY | 1.514 | <1×10^-5^ | 9.09×10^-5^ |
| KEGG_TOLL_LIKE_RECEPTOR_SIGNALING_PATHWAY | 1.511 | <1×10^-5^ | 8.48×10^-5^ |
| KEGG_EPITHELIAL_CELL_SIGNALING_IN_HELICOBACTER_PYLORI_INFECTION | 1.504 | <1×10^-5^ | 7.71×10^-5^ |
| KEGG_TGF_BETA_SIGNALING_PATHWAY | 1.499 | <1×10^-5^ | 9.70×10^-5^ |
| KEGG_NOD_LIKE_RECEPTOR_SIGNALING_PATHWAY | 1.497 | <1×10^-5^ | 1.18×10^-4^ |

NES, normalized enrichment score; NOM, nominal; FDR, false discovery rate.
